# EBNA2 Drives Formation of New Chromosome Binding Sites and Target Genes for B-Cell Master Regulatory Transcription Factors RBP-jκ and EBF1

**DOI:** 10.1371/journal.ppat.1005339

**Published:** 2016-01-11

**Authors:** Fang Lu, Horng-Shen Chen, Andrew V. Kossenkov, Karen DeWispeleare, Kyoung-Jae Won, Paul M. Lieberman

**Affiliations:** 1 The Wistar Institute, Philadelphia, Pennsylvania, United States of America; 2 The Institute for Diabetes Obesity and Metabolism, Department of Genetics, Perelman School of Medicine, University of Pennsylvania, Philadelphia, Pennsylvania, United States of America; Tulane Health Sciences Center, UNITED STATES

## Abstract

Epstein-Barr Virus (EBV) transforms resting B-lymphocytes into proliferating lymphoblasts to establish latent infections that can give rise to malignancies. We show here that EBV-encoded transcriptional regulator EBNA2 drives the cooperative and combinatorial genome-wide binding of two master regulators of B-cell fate, namely EBF1 and RBP-jκ. Previous studies suggest that these B-cell factors are statically bound to target gene promoters. In contrast, we found that EBNA2 induces the formation of new binding for both RBP-jκ and EBF1, many of which are in close physical proximity in the cellular and viral genome. These newly induced binding sites co-occupied by EBNA2-EBF1-RBP-jκ correlate strongly with transcriptional activation of linked genes that are important for B-lymphoblast function. Conditional expression or repression of EBNA2 leads to a rapid alteration in RBP-jκ and EBF1 binding. Biochemical and shRNA depletion studies provide evidence for cooperative assembly at co-occupied sites. These findings reveal that EBNA2 facilitate combinatorial interactions to induce new patterns of transcription factor occupancy and gene programming necessary to drive B-lymphoblast growth and survival.

## Author Summary

Epstein-Barr Virus (EBV) reprograms host cell transcription through multiple mechanisms. Here, we show that EBV-encoded transcriptional co-activator EBNA2 drives the formation of new chromosome binding sites for host cell factors RBP-jκ and EBF1. The formation of these new sites is EBNA2-dependent. These newly formed sites have overlapping or neighboring consensus binding sites for these factors, but are only co-occupied in the presence of EBNA2. Newly formed, co-occupied binding sites are highly enriched at promoter and enhancer regulatory elements of genes activated by EBV and required for B-cell proliferation and survival. These findings indicate that EBNA2 drives cooperative and combinatorial transcription factor interactions on chromosomal DNA. We suggest that models depicting the static binding of master regulatory transcription factors to consensus binding sites be revised, and that co-activators, like EBNA2, induce dynamic and combinatorial selection of genome-wide binding sites to alter gene regulation.

## Introduction

Tumor viruses encode many factors that mimic and alter host cell processes. Epstein-Barr Virus (EBV) is a human tumor virus associated with various lymphoid and epithelial cell malignancies, including Burkitt’s lymphoma, nasopharyngeal carcinoma, and lymphoproliferative disorders in the immunosuppressed [1, 2]. EBV can efficiently immortalize naïve B-lymphocytes and establish long-term latent infection in these immortalized cells [3]. EBV expresses different viral gene products depending on the cell or tumor type where latent infection is established [4]. During natural infection, EBV drives naïve resting B-cells into a proliferative program resembling antigen driven B-cell germinal center reaction [5]. EBV drives B-cell proliferation and differentiation through a complex combination of viral proteins and non-coding RNAs [4]. EBV gene expression programs also change coordinately with the differentiating host-cell, and these changes have been referred to as latency types [5]. Thus, EBV infection mimics B-cell developmental programs in the absence of normal exogenous antigenic signal [5].

EBV encodes several nuclear proteins that modulate host and viral gene transcription [6]. EBNA2 is potent transcriptional co-activator that is expressed in early stages of EBV-induced proliferation of naïve B-cells, but its expression is extinguished at later stages of B-cell development. EBNA2 is best characterized for its physical interaction with the sequence-specific transcription factors RBP-jκ (also called RBPJ, CBF1, and CSL) at promoters and enhancers of EBNA2-regulated genes ([7, 8](reviewed in [9, 10]). RBP-jκ is thought to bind constitutively to many of transcriptional regulatory elements, and function as a scaffold for co-activators, like intracellular Notch, or various co-repressors in the absence of Notch [11]. While EBNA2 and Notch are not interchangeable for either viral or cellular functions, EBNA2 may be considered a viral mimic of Notch at some transcriptional regulatory elements. Recent genome-wide studies have revealed that EBNA2 frequently colocalizes with RBP-jκ, as well as other cellular factor binding sites, including Early B-cell Factor 1 (EBF1) [12]. EBF1 is a sequence specific DNA binding protein that functions as a B-cell identity factor necessary for the establishment of pro-B cells and maintenance of B-cell specific transcription programs [13]. EBNA2 colocalizes with EBF1 and RBP-jκ at many enhancer and super-enhancer regions in EBV positive lymphoblastoid cell lines [14]. Both EBF1 and RBP-jκ are master regulatory transcription factors that play fate determining roles in lymphoid cell development, but their functional cooperativity has not been experimentally established. Moreover, EBNA2 is not thought to alter the chromosome binding site selection of these sequence specific DNA binding factors.

Patterns of transcription factor binding to chromosomal regulatory elements is a primary determinant of cell fate and identity [13, 15, 16]. Transcription programs depend on many intrinsic and extrinsic factors that converge ultimately on the selective binding of transcription factors to recognition elements that may be embedded in non-permissive chromatin environments and subject to repressive epigenetic modifications. In addition to overcoming chromatin and epigenetic barriers, transcription factors engage in complex combinatorial interactions at promoter and enhancer regulatory elements. How combinatorial interactions between DNA-binding and non-binding factors contribute to site selection and transcription programming in a genome-wide context remains largely unexplored. Here we investigate how a viral-encoded non-DNA binding coactivator, EBNA2, can reprogram the target binding sites and functional cooperativity of two host cell master regulatory transcription factors, EBF1 and RBP-jκ.

Transcription factors with sequence-specific DNA binding capability are known to recognize a distinct primary DNA sequence element. However, not all consensus sequences are occupied by these transcription factors. Epigenetic factors, including DNA methylation, nucleosome positioning, heterochromatin, and competitive transcription factor binding may influence the occupancy at different chromosomal locations in cell-type dependent manners. It is also known that some chromosome occupancy may depend on cooperative binding with additional factors, including sequence specific factors and non-DNA binding co-regulatory factors. Here, we present evidence that EBNA2 is one such co-regulatory factor that alters the genome wide distribution of sequence-specific B-cell master regulatory factors EBF1 and RBP-jκ.

## Results

### EBF1 and RBP-jκ binding to EBV genomes vary in different latency types

**Fig 1:**
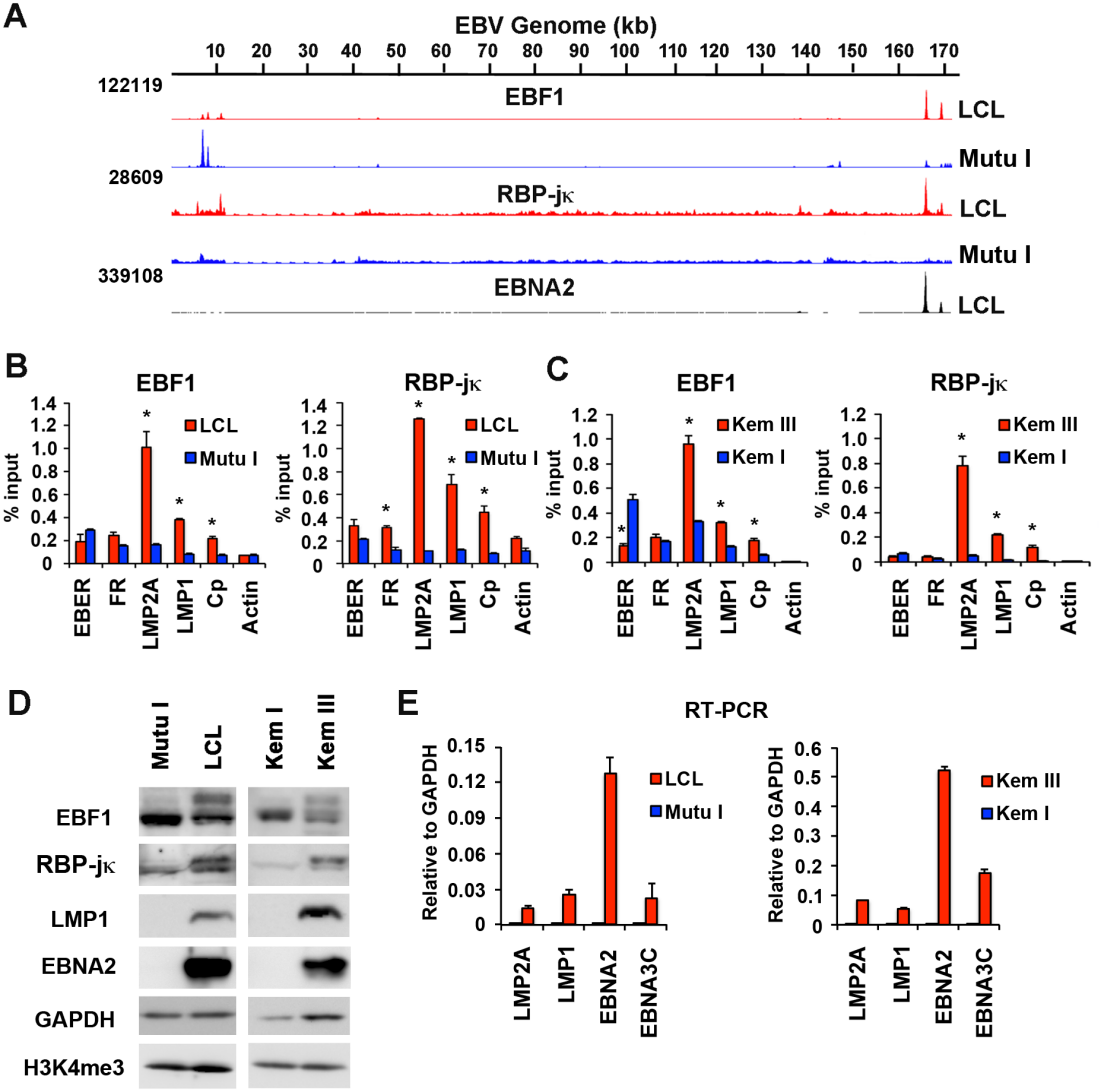
Differential binding of transcription factors on EBV genomes in type I and type III latency. **(A)** ChIP-Seq tracks mapped to EBV genome in LCL (red) or Mutu I (blue) for EBF1 or RBP-jκ as indicated. EBNA2 ChIP-Seq for LCL (black). Y-axis represent raw read counts. **(B)** ChIP-qPCR for EBF1 or RBP-jκ in LCL (red) or Mutu I (blue) at various EBV genome regulatory regions as indicated and Actin genomic region as negative control. **(C)** Same as in B, except for isogenic EBV positive BL cells Kem III (red) or Kem I (blue). Asterisk indicates p < 0.05. **(D)** Western blot of Mutu I, LCL, Kem I, and Kem III probed with antibody to EBF1, RBP-jκ, LMP1, and EBNA2, with cellular loading controls for GAPDH and H3K4me3, as indicated. **(E)** RT-qPCR for EBV type III gene expression in LCL or Mutu I (left panel) or Kem I and Kem III (right panel).

To investigate the landscape of transcription factor binding on identical genomes in two closely related cell types, we first compared the binding patterns of EBF1 and RBP-jκ on the EBV genomes in B-cells with distinct latency types. We compared an EBV positive lymphoblastoid cell line (LCL) with a type III latency expression pattern (EBNA2 positive) to an EBV positive Burkitt lymphoma (BL) cell line (Mutu I) with a type I latency expression pattern (EBNA2 negative) (Figs 1A, 1B, 1C and S1). We found that EBF1 and RBP-jκ bound with very similar occupancy in LCLs, but with different binding patterns in Mutu I. In LCL, EBF1 and RBP-jκ bound in close proximity to each other at the promoter regulatory elements for LMP1 and LMP2A. In contrast, neither EBF1 nor RBP-jκ binding was observed at the LMP1 and LMP2A locus in Mutu I. Both LMP1 and LMP2A are expressed at high levels in LCLs, but are repressed in Mutu I cells (Fig 1D and 1E). Mutu I-selective enrichment of EBF1 was observed at the EBER and OriP control region, which has important transcription regulatory functions in type I cells [17]. The LCL-specific enrichment of EBF1 and RBP-jκ binding at LMP1 and LMP2A was confirmed by ChIP-qPCR (Fig 1B). We also show that this general pattern of EBF1 and RBP-jκ occupancy is observed in an isogenic BL with type I (Kem I) or type III (Kem III) pattern of viral latency gene expression (Fig 1C). Protein expression of EBF1 and RBP-jκ, as well as EBV proteins specific for type III latency, were monitored by Western blot (Fig 1D). Although some differences in protein expression levels were observed, these differences alone are unlikely to account for the global changes in chromosome occupancies. We also observed some changes in EBF1 and RBP-jκ protein mobility, suggesting that post-translational modifications or alternative isoforms may contribute to the different chromosome occupancy. As expected, EBV type III latency genes were expressed exclusively in type III cells (Fig 1E). Taken together, these findings suggest that EBF1 and RBP-jκ can have cell type-dependent DNA binding patterns.

### Cell-type variations in EBF1 and RBP-jκ chromosome binding sites

**Fig 2:**
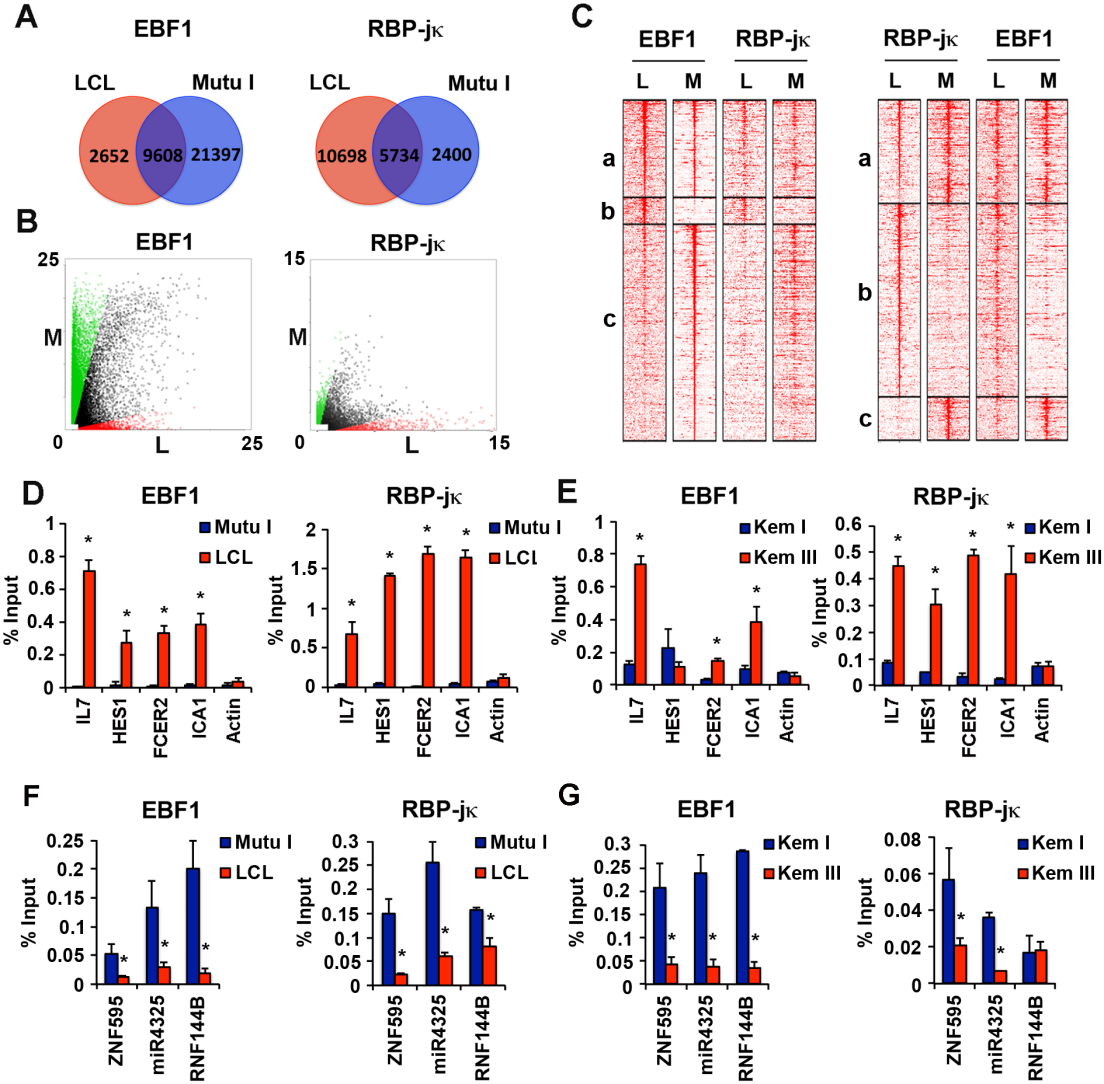
Differential binding of transcription factors on cellular genome in type I and type III infected B-lymphocytes. **(A)** Venn diagram showing EBF1 and RBP-jκ occupancy sites specific to LCL (red) and Mutu I (blue) or common to both cell types. **(B)** Scatter plot showing the distribution of EBF1 (left) or RBP-jκ (right) occupancies that are specific to either LCL (L, red) or Mutu I (M, green) or common to both cell types (grey). **(C)** Heatmaps showing co-occupancy of EBF1 and RBP-jκ from either LCL (L) or Mutu I (M) cells, centered on either EBF1 in LCL (left panel) or RBP-jκ in LCL (right panel). Binding sites were clustered based on their common occurrence in MutuI and LCL (a), LCL-specific (b), or Mutu I-specific (c). **(D and F)** ChIP-qPCR validation for various cellular gene ChIP-Seq peaks for EBF1 (left) or RBP-jκ (right) in either Mutu I (blue) or LCL (red) cells. **(E)** Same as in **(D)** except for Kem I and Kem III cell types. **(G)** Same as in **(F)** except for Kem I and Kem III cell types. Asterisk indicates p < 0.05.

To determine if the cell-type specific binding of EBF1 and RBP-jκ extended to the cellular genome, we compared ChIP-Seq data for cellular genome binding patterns in LCL and Mutu I cells (Fig 2). We found that both EBF1 and RBP-jκ had large numbers of cell-type dependent binding sites, as well as a subset of binding sites common to both cells (Fig 2A). Total and cell-type specific EBF1 binding sites were lower in LCL relative to Mutu I, while RBP-jκ binding sites showed the inverse trend. The peak intensities of RBP-jκ and EBF1 binding sites also correlated with cell-type specific distribution as shown by scatter plot (Fig 2B). A heat map of binding sites centered on EBF1 site (Fig 2C left panels), or RBP-jκ sites (Fig 2C right panels) shows the relative distribution and co-occupancies of EBF1 and RBP-jκ in the different cell types. We observed that RBP-jκ sites trended to colocalize with EBF1. While the overlap of EBF1 and RBP-jκ binding was not restricted to LCLs, a distinct cluster of colocalized sites could be assigned as specific to each cell type. ChIP-Seq data sets were analyzed for localization to nearest gene transcription start sites, and these sites were then selected and retested for their cell-type specific enrichment by ChIP-qPCR (Fig 2D). Several of the co-occupied LCL-specific sites were found at well-established RBP-jκ regulated genes, including IL7, HES1, FCER2, and ICA1. These showed strong and selective enrichment for both EBF1 and RBP-jκ in LCL relative to Mutu I. In contrast, ZNF595, miR4325, and RNF144B showed Mutu I-specific co-occupancy (Fig 2F). We also examined the binding patterns of RBP-jκ and EBF1 in the isogenic BL-cells Κem I and Kem III, that have different EBV latency type gene expression programs (Fig 2E and 2G). EBF1 and RBP-jκ were highly enriched at the IL7, FCER2, and ICA1 gene loci in Kem III, but not Kem I (Fig 2E). In contrast, ZNF595, miR4325, and RNF144B were enriched for EBF1 and RBP-jκ in Kem I, but not Kem III (Fig 2G). HES1 was enriched for RBP-jκ in Kem III, but not for EBF1, indicating there are some cell type specific differences between Kem III and LCLs. Nevertheless, these findings suggest that master regulatory transcription factors, like RBP-jκ and EBF1, have different genomic binding patterns in different cell types and this appears to correlate with latency type gene programming.

### EBF1 and RBP-jκ co-occupy a subset of EBNA2 binding sites

**Fig 3:**
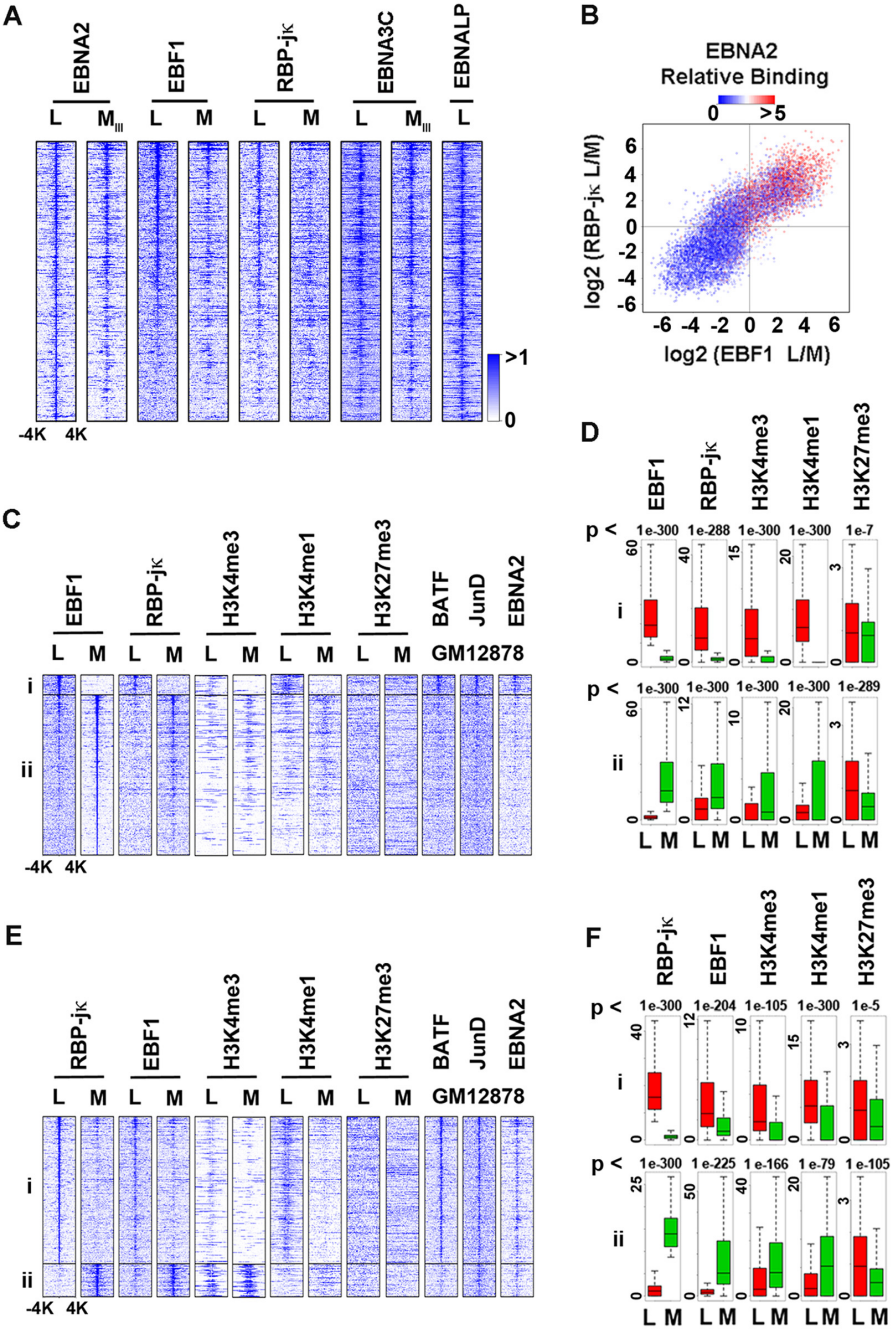
Colocalization of EBNA2 with RBP-jκ and EBF1 co-occupied sites. **(A)** Heatmap of EBNA2 ChIP-Seq peaks from LCL was compared with EBNA2 peaks from Mutu III, EBF1 and RBP-jκ from LCL or Mutu I, EBNA3C from LCL or Mutu III, or EBNALP from LCL. Peaks were sorted based on the EBNA2 levels in LCL and show -/+ 4kb window. **(B)** The fold changes (LCL versus Mutu I) were plotted for EBF1 (x-axis) and RBP-jκ (y-axis) for all EBF1 and RBP-jκ peaks. The peaks were colored based on the EBNA2 occupancy. The positive correlation (Pearson’s correlation coefficient = 0.8) between the fold changes suggests that EBF1 and RBP-jκ co-localize with each other. The strong occupancy of EBNA2 at the top-right panel of the plot shows that high occupancy EBNA2 correlates with sites of high occupancies for EBF1 and RBP-jκ. **(C**) Heatmap comparison of histone modification marks (H3K4me3, H3Km4me1, H3K27me3) and transcription factors BATF, JunD, and EBNA2 from LCL centered at EBF1 peaks enriched in LCL (cluster i) or Mutu I (cluster ii). **(D)** Box plot quantitation of peak number and intensity for ChIP-Seq clustered sets i and ii shown in panel C as indicated. **(E and F)** Same as in C and D, except centered at cell-specific RBP-jκ peaks.

EBV-encodes several transcriptional co-regulators that binds RBP-jκ and are expressed in type III, but not type I latency. We therefore aligned our ChIP-Seq data sets for RBP-jκ and EBF1 with several published ChIP-Seq data sets for EBNA2, EBNA3C, and EBNALP from both LCL and Mutu III cells [12, 18–20] (Fig 3A). The de novo motif analysis on EBNA2 peaks from LCL identified RBP-jκ, as well as EBF1 as significant motifs (S2 Fig). Aligning these data sets with EBNA2 from LCL revealed strong correlations with EBNA2 from Mutu III, as well as EBNA3C and EBNALP, as previously reported. Analysis of EBF1 and RBP-jκ ChIP-Seq revealed significant colocalization with EBNA2 in LCLs, but not in Mutu I (Figs 3A and S3). To further investigate the correlation of EBNA2 peaks with the LCL-specific EBF1 and RBP-jκ binding sites, we investigated the ratio of the peak intensity for EBF1 (x-axis) and RBP-jκ (y-axis) between LCL and Mutu I (Fig 3B). The ratio of EBF1 and RBP-jκ peak intensity (L/M) was highly correlated with EBNA2 co-occupancy (correlation coefficient = 0.8), suggesting that EBNA2 trends towards EBF1 and RBP-jκ sites that are of greater peak intensity in LCLs relative to Mutu I. These findings indicate that EBNA2 binds frequently at EBF1 and RBP-jκ co-occupied sites, and that these sites are enriched in LCL relative to a BL cell line with type I latency.

### Enhancer-like features of EBF1-RBPjκ-EBNA2 co-occupied sites

The convergent binding of EBNA2, EBF1, and RBP-jκ was further analyzed for colocalizations with other well-established histone modifications and transcription factors (Fig 3C–3F). We generated new ChIP-Seq data sets for histone modifications H3K4me3, H3K4me1, and H3K27me3, and combined it with existing published data sets for transcription factors BATF and JunD [21], because the motif for AP1 factor (TGAnTCA) was significantly observed in the de novo motif finding results (S2 Fig). We also oriented the analysis centered over the EBF1 (Fig 3C and 3D) or RBP-jκ (Fig 3E and 3F) binding sites in LCLs. Several conclusions could be drawn from these analyses. First, the LCL-specific binding sites (cluster i) for EBF1 colocalize with RBP-jκ and EBNA2, and are highly enriched for H3K4me1, as well as BATF and JunD (Fig 3C and 3D). This suggests that these LCL-specific sites have enhancer-like properties since they are highly enriched in H3K4me1, a well characterized mark of transcriptional enhancers [22]. EBF1 sites that are highly enriched in Mutu I (cluster ii) also colocalize with Mutu I RBP-jκ, but are less enriched for EBNA2, BATF, and JunD, and have reduced H3K4me1 in LCLs, while H3K4me3 remains enriched in Mutu I co-occupied sites. This suggests that cluster ii sites colocalize with transcriptional start sites, and less commonly with enhancer elements. When the analysis was centered on RBP-jκ binding sites (Fig 3E and 3F), we observed strong co-localization of RBP-jκ with EBF1 in both LCL (cluster i) and Mutu I (cluster ii) enriched sites. LCL-specific sites (cluster i) were highly enriched for H3K4me1, BATF, and JunD. Mutu I specific sites (cluster ii) were highly enriched for H3K4me3, suggesting that these sites colocalize at transcription start sites. Taken together, these findings indicate that RBP-jκ and EBF1 have functionally distinct interactions at different locations in a cell-type specific manner, and that EBNA2 strongly colocalizes with enhancer-like histone modification H3K4me1 in LCL.

### EBF1-specific binding correlates with transcription activity

**Fig 4:**
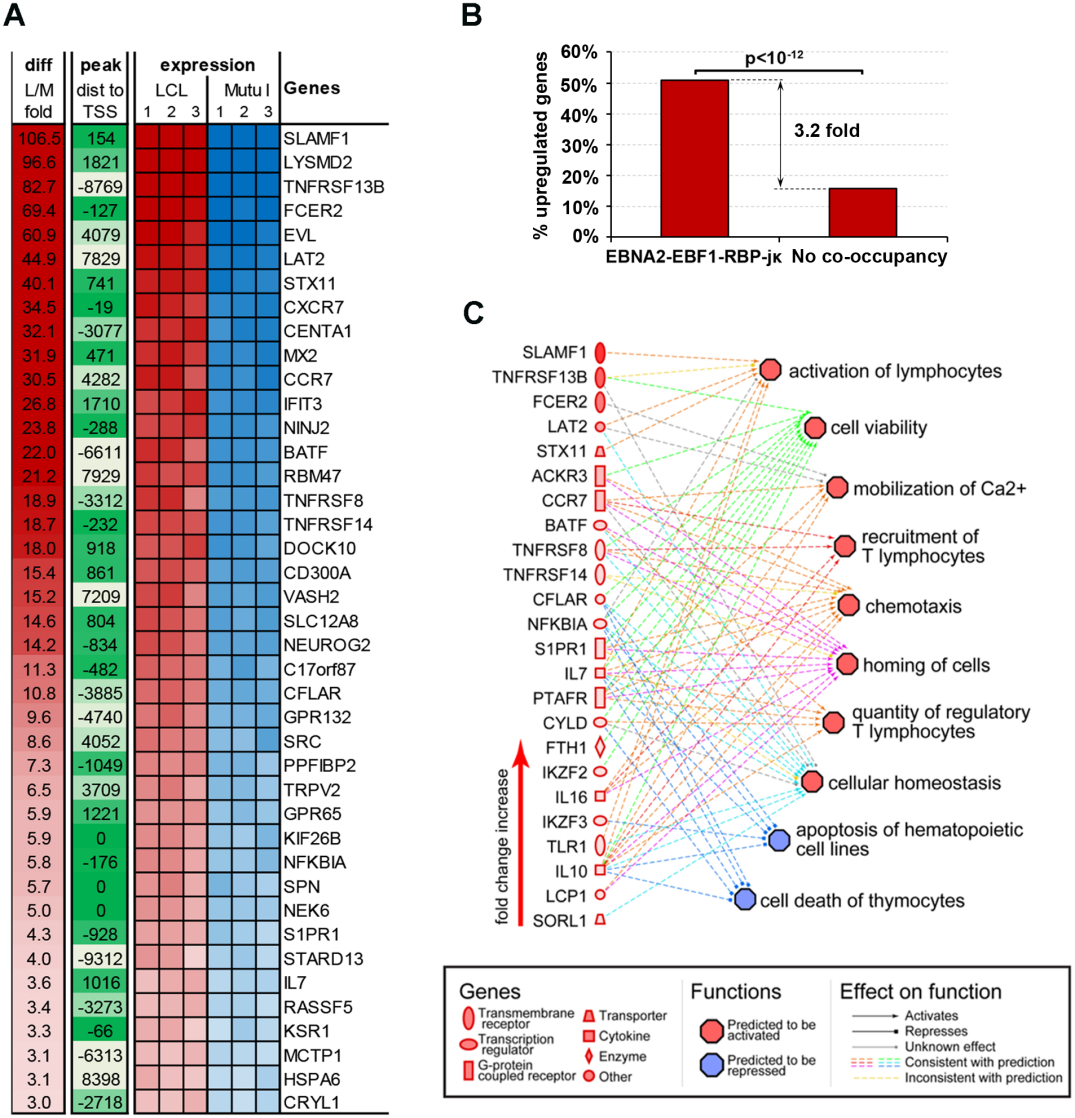
Characterization of cellular genes highly activated by EBF1-RBP-jκ-EBNA2 co-occupancy in LCL. **(A)** Heatmap of mRNA expression for genes with co-localized EBF1, RBP-jκ, and EBNA2 and at least 3-fold over expression in LCL relative to Mutu I. **(B)** Percentage of genes with co-localized EBF1, RBP-jκ, and EBNA2 sites at least 10kb from the TSS that are over-expressed in LCLs relative to Mutu I. **(C)** Predicted regulation network for LCL over-expressed genes co-occupied by EBNA2, EBF1, and RBP-jκ.

To investigate the functional significance of the co-occupancy of EBNA2 with EBF1 and RBP-jκ, we analyzed the mRNA expression of LCL and Mutu I cells using Illumina microarrays (Figs 4 and S4). We focused on genes that had transcription start sites within 10 kb of an EBNA2-EBF1-RBP-jκ co-occupied binding site in LCLs (Fig 4A). We identified 165 such genes, and observed that 84 of those genes were significantly up-regulated in LCL compared to Mutu I, an enrichment of 3.2 fold more than expected by random chance (p<10^−12^ by Fisher exact test, Fig 4B). Ingenuity Pathway analysis of these upregulated genes revealed a network of important B-lymphocyte functions, including activation of lymphocytes, mobilization of calcium, cell viability, and chemotaxis (Fig 4C).

### EBNA2-dependent DNA-binding of EBF1 and RBP-jκ co-occupied chromosomal sites

**Fig 5:**
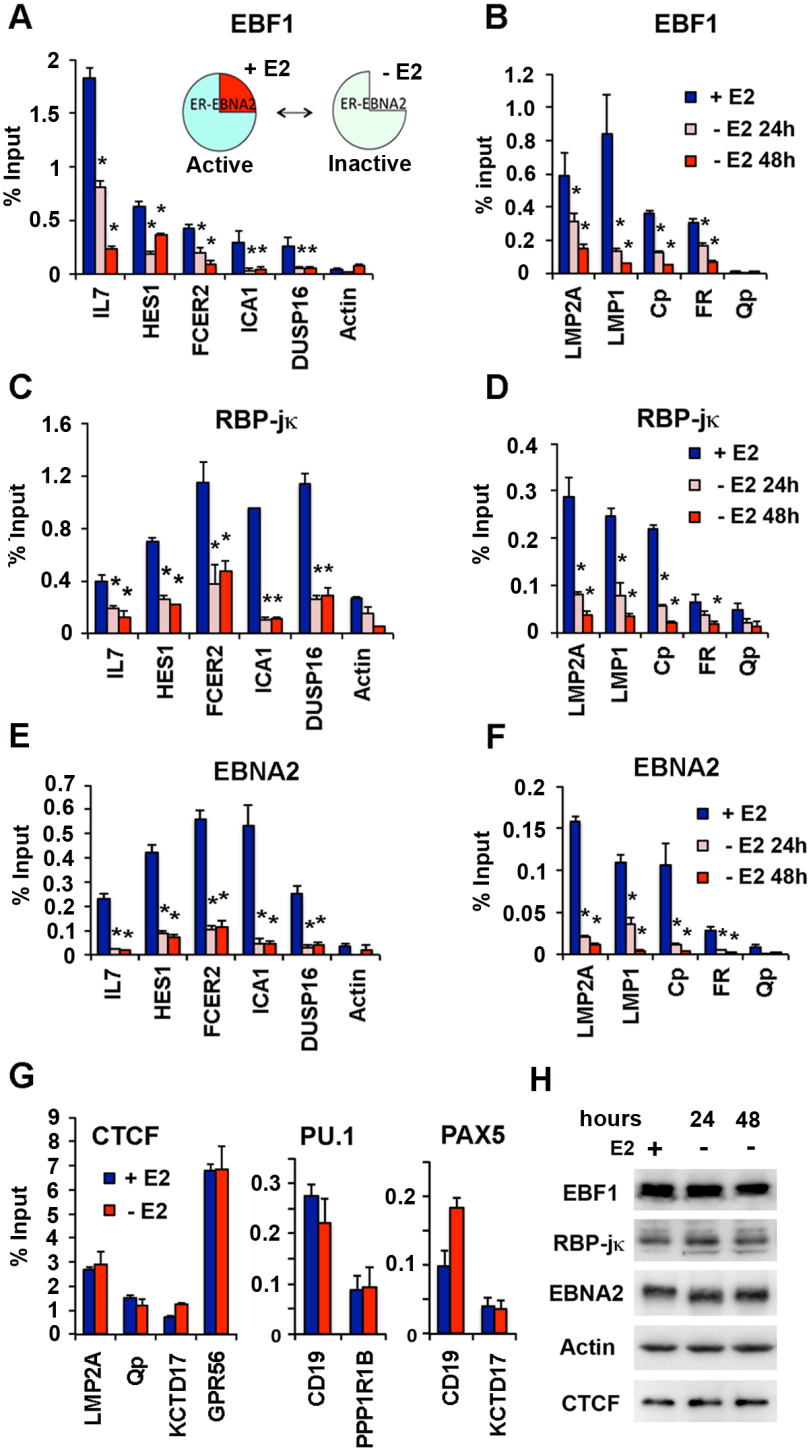
EBNA2-dependent transcription factor redistribution in chromatin occupancy. EREB2.5 cells were treated with (+) or without (-) estradiol (E2) for 24 or 48 hrs and then assayed by ChIP for binding to EBF1 (**A** and **B**), RBP-jκ (**C** and **D**), or EBNA2 (**E** and **F**) at cellular (**A, C, E**) or EBV genome sites (**B, D, F**). Actin genomic region (cellular) or Qp (EBV) was used as negative binding control for EBF1, RBP-jκ, ορ EBNA2 ChIP. **(G)** ChIP binding for CTCF, PU.1, or PAX5 in EREB2.5 cells treated (blue) or untreated (red) with E2 for 48 hrs. PPP1R1B or KCTD17 genomic region was negative binding control PU.1 or PAX5, respectively. Asterisk indicates p < 0.05. **(H)** Western blot showing protein levels for EBF1, RBP-jκ, EBNA2, Actin, and CTCF in EREB2.5 cells at 24 and 48 hrs after E2 withdrawal.

Correlations studies suggest that EBNA2 may facilitate binding and co-occupancy of RBP-jκ and EBF1 in LCLs. To experimentally determine if EBNA2 can alter EBF1 and RBP-jκ chromosome occupancy, we utilized an EBNA2-inducible cell line, EREB2.5, that contains an EBNA2 protein fused to the estrogen receptor (ER) [23]. The ER-EBNA2 fusion in EREB2.5 cells regulates the nuclear-cytoplasmic transport of EBNA2 and therefore has a rapid post-translational effect on EBNA2 function in the nucleus. Upon withdrawal of estrogen and inactivation of EBNA2, we found a rapid loss of binding for EBF1 and RBP-jκ at sites that colocalize with EBNA2 (Fig 5A–5D). As expected, EBNA2 binding was rapidly eliminated from all sites tested (Fig 5E and 5F). EBF1 binding was not disrupted at most sites that were not overlapping with EBNA2 binding sites, while RBP-jκ binding was modestly affected at some of sites that were not overlapping with EBNA2 sites (S5A, S5B and S5C Fig). In the EBV genome, we observed a strong EBNA2-dependence of EBF1 and RBP-jκ binding to LMP2A, LMP1, and C promoter regulatory elements (Fig 5B and 5D). Estradiol withdrawal did not have similar inhibitory effects on binding of CTCF, PU.1, or PAX5 (Fig 5G), suggesting that these effects are selective for the EBNA2 interacting proteins RBP-jκ and EBF1. Western blot analysis indicated that protein levels for EBF1 and RBP-jκ were not significantly altered upon estradiol withdrawal, indicating that loss of protein expression is not the mechanism for the altered binding site selectivity (Fig 5H). Although estradiol withdrawal induced a G1 cell cycle arrest at 48 hrs (S6 Fig), the re-addition of estradiol after 72 hrs withdrawal rescued EBF1 and RBB-jκ binding to their co-occupied cellular target sites (S7 Fig). These findings indicate that EBNA2 is necessary and sufficient to maintain the stable binding of EBF1 and RBP-jκ at co-occupied sites in LCLs.

**Fig 6:**
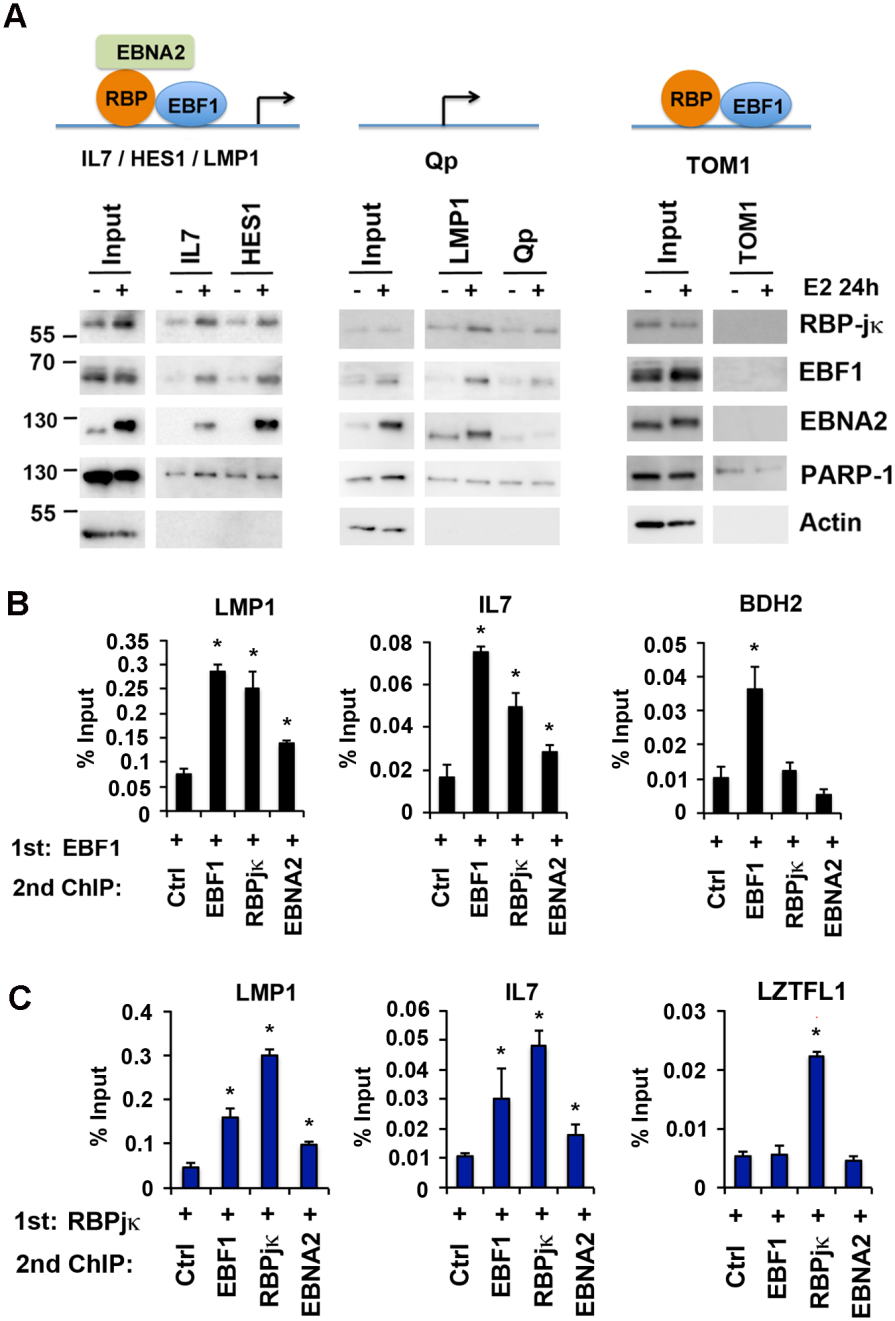
Cooperative binding and colocalization of EBF1, RBP-jκ, and EBNA2 at DNA regulatory elements. **(A)** DNA-affinity binding assays with nuclear extracts from EREB2.5 cells with (+) or without (-) estradiol (E2) using DNA regulatory elements from IL7, HES1 (left), or LMP1, Qp (middle), or TOM1 (right). Input (10%) is indicated, and bound proteins were assayed by Western blot for EBF1, RBP-jκ, EBNA2, and specificity controls for PARP1 and Actin. **(B)** ChIP-reChIP assays in LCLs with EBF1 as first ChIP, followed by a reChIP with either no antibody control or antibody to EBF1, RBP-jκ or EBNA2. ReChIP DNA was quantified by qPCR at LMP1, IL7, and BDH2 (an EBF1 only site) binding sites. **(C)** Same as in B, except first ChIP was with RBP-jκ followed by a ReChIP with no antibody control, or EBF1, RBP-jκ, or EBNA2 antibody and assayed at LMP1, IL7, or LZTFL1 (an RBP-jκ only site) binding sites. Asterisk indicates p < 0.05.

To explore the biochemical basis for the altered DNA binding properties of EBF1 and RBP-jκ in the presence of EBNA2, we first performed co-IP experiments (S8 Fig). We confirmed that EBNA2 can co-IP with RBP-jκ, as expected, but did not find any evidence for a stable interaction between EBF1 with either RBP-jκ or with EBNA2 (S8 Fig). We next tested whether EBNA2 may facilitate EBF1 and RBP-jκ DNA binding using *in vitro* DNA affinity assays followed by Western blotting (Fig 6A). We compared extracts derived from EREB2.5 cells treated with or without estradiol for their ability to bind to several different DNA elements, including those with closely overlapping EBF1 and RBP-jκ sites that colocalize with EBNA2 (IL7, HES1, and LMP1) (S9 Fig), as well as negative controls lacking EBNA2 binding sites (Qp and TOM1). We observed that nuclear extracts derived from E2 treated EREB2.5 cells were enriched for EBNA2 protein and bound EBNA2 efficiently in DNA affinity assays. We found that EBF1 and RBP-jκ binding were enhanced in the presence of EBNA2 at the IL7, HES1, and LMP1 DNA elements, but not at Qp or TOM1 DNA controls. The failure to bind TOM1 was surprising, since EBF1 and RBP-jκ can bind in EREB cells in the absence of EBNA2. Experimental conditions were optimized for EBNA2 binding, and these conditions may limit detection of weaker interactions that occur at sites that lack EBNA2 colocalization. The non-specific DNA binding protein PARP1 bound independently of ER treatment, while the non-DNA binding protein Actin did not bind DNA templates, providing some control for specificity. These findings suggest that EBNA2 protein can partially facilitate EBF1 and RBP-jκ binding at some DNA elements, but that other factors, perhaps chromatin structure and epigenetic modifications, must contribute substantially to the cell-type selective binding observed in living cells.

To determine if EBF1 and RBP-jκ bind simultaneously to the same DNA elements, we performed ChIP-reChIP assays (Fig 6B and 6C). We compared LMP1 and IL7 promoter regions that have strong overlapping co-occupied sites for EBF1, RBP-jκ and EBNA2. We found that ChIP-reChIP of EBF1 primary ChIPs with either EBF1, RBP-jκ, or EBNA2 resulted in significant enrichment at LMP1 and IL7, suggesting that these proteins form complexes on the same DNA elements. The BDH2 gene has an EBF1 site that is not co-occupied by RBP-jκ or EBNA2 in ChIP-Seq data, and showed no significant enrichment when subject to reChIP (Fig 6B, right panel). Similarly, when RBP-jκ was used as the primary ChIP, reChIP with either EBF1, RBP-jκ, or EBNA2 showed significant enrichment at the LMP1 and IL7 promoters, but not at the LZTFL1 gene which has an RBP-jκ site that lacks EBF1 or EBNA2 co-occupancy in ChIP-Seq data sets (Fig 6C). These findings suggest that EBNA2, RBP-jκ, and EBF1 can form a stable complex simultaneously on the same DNA elements that where they colocalize in ChIP-Seq experiments.

### Functional requirement for both EBF1 and RBP-jκ at sites co-occupied by EBNA2

**Fig 7:**
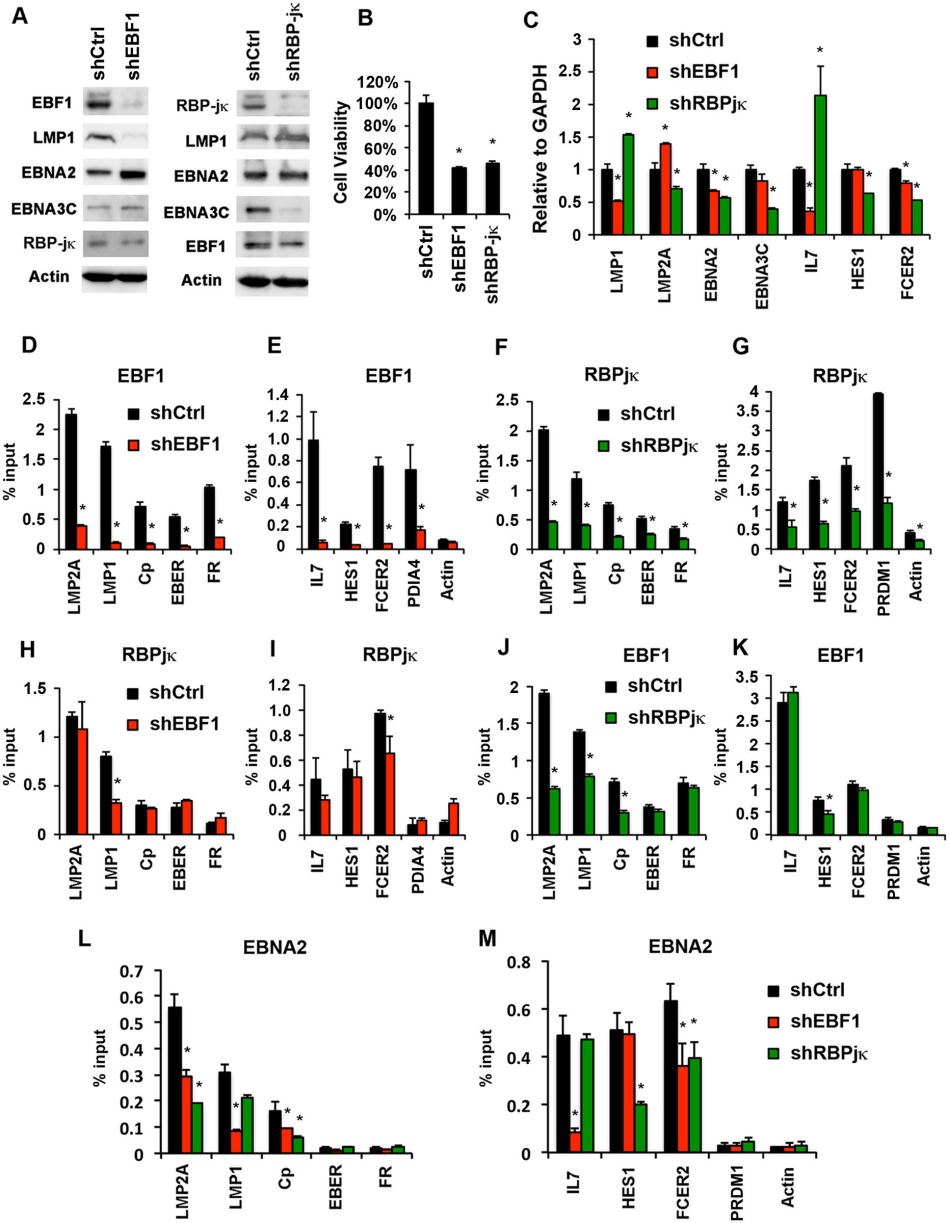
EBF1 and RBP-jκ function at EBNA2 co-occupied sites. **(A)** Western blots of LCL cells transduced with either control shRNA (shCtrl) or shEBF1 (left) or shCtrl or shRBP-jκ (right) lentivirus and probed for EBF1, RBP-jκ LMP1, EBNA2, EBNA3C, or Actin. **(B)** Cell viability determined by Resazurin assay for LCL cells 7 days after shRNA lentivirus transduction. **(C)** RT-qPCR (ΔΔCT) analysis of EBV or cellular gene transcription (as indicated) in LCLs transduced with either shEBF1 (red), shRBP-jκ (green), or shCtrl lentivirus (black). **(D-M)** ChIP-qPCR in LCLs with antibodies to either EBF1 (panels **D**, **E**, **J**, **K**), RBP-jκ (panels **F**, **G**, **H**, **I**), or EBNA2 (panels **L** and **M**). LCLs were transduced with either shCtrl (black), shEBF1 (red), or shRBP-jκ (green). Viral (panels **D, F, H, J, L**), or cellular (**E, G, I, K, M**) sites were analyzed as indicated. Asterisk indicates p < 0.05.

To determine whether EBF1 or RBP-jκ may contribute to transcription regulation at some of these co-occupied sites, we used shRNA depletion of either EBF1 or RBP-jκ in LCL cells (Fig 7). Both EBF1 and RBP-jκ shRNA led to an efficient depletion of their respective targets, as determined by Western blot (Fig 7A). Interestingly, EBF1 depletion led to a loss of LMP1 protein expression from LCLs, but no loss of other type III proteins, including EBNA2 or EBNA3C. In contrast, RBP-jκ depletion led to a loss of EBNA3C, but no loss EBNA2 or LMP1. This suggests that EBF1 and RBP-jκ have non-redundant functions in the transcription regulation of EBV type III gene expression. Both LMP1 and EBNA3C are essential for LCL viability, and as expected, both EBF1 and RBP-jκ depletion decreased LCL cell viability (Fig 7B). RT-qPCR analysis confirmed that EBF1 shRNA depletion led to a loss of LMP1 transcription, while RBP-jκ led to loss of EBNA3C (Fig 7C). RT-PCR also revealed several other changes in EBV gene expression in response to EBF1 or RBP-jκ depletion, including a modest decrease in EBNA2 expression. Examination of cellular gene mRNA revealed some selective sensitivity to either EBF1 or RBP-jκ depletion. IL7, like LMP1, was more sensitive to EBF1 depletion, while HES1 was more sensitive to RBP-jκ depletion. FCER2, like EBNA2, was similarly affected by either EBF1 or RBP-jκ (Fig 7C). ChIP assays revealed a strong loss of EBF1 from shEBF1 depleted cells (Fig 7D and 7E), and RBP-jκ from RBP-jκ depleted cells (Fig 7F and 7G) at all viral and cellular binding sites tested. Interestingly, in shEBF1 depleted cells, RBP-jκ binding was reduced at the LMP1 promoter (Fig 7H) and at the IL7 and FCER2 cellular promoters (Fig 7I). On the other hand, in shRBP-jκ depleted cells, EBF1 binding was reduced at the LMP2A, LMP1, and Cp viral promoters (Fig 7J) and HES1 cellular promoter (Fig 7K). These findings suggest that some, but not all EBF1 and RBP-jκ binding sites are interdependent. Finally, we examined EBNA2 binding in shEBF1 or shRBP-jκ depleted cells (Fig 7L and 7M). We found that EBNA2 binding was reduced at LMP2A, LMP1, and Cp viral promoters, and IL7 and FCER2 cellular promoters in shEBF1 depleted cells. On the other hand, in shRBP-jκ depleted cells, we found that EBNA2 binding was reduced at LMP2A, Cp, and cellular HES1. These findings suggest that EBNA2 may be recruited to some sites by either RBP-jκ or EBF1, or both. Taken together, these findings indicate that EBF1 and RBP-jκ have non-redundant, cooperative and context-dependent functions at viral and cellular promoters where they colocalize with EBNA2.

## Discussion

A fundamental unresolved question in eukaryotic gene regulation is how transcription factors find their appropriate cell-type specific binding sites in the context of chromatin and other epigenetic parameters. Pioneering factors, like EBF1, are thought to have the intrinsic capacity to access their chromatin-embedded binding sites to form new promoter and enhancers elements necessary for cell-specific gene transcription [15]. While this is well-established for many genomic locations, our findings provide evidence that a significant number of transcription factor binding sites require cooperative interactions with neighboring factors and accessory co-factors. Our findings indicate that transcriptional cofactors, like EBNA2, can function as pioneer factor ‘scouts’ that reorient chromosome occupancy for client pioneer proteins, like RBP-jκ and EBF1.

**Fig 8:**
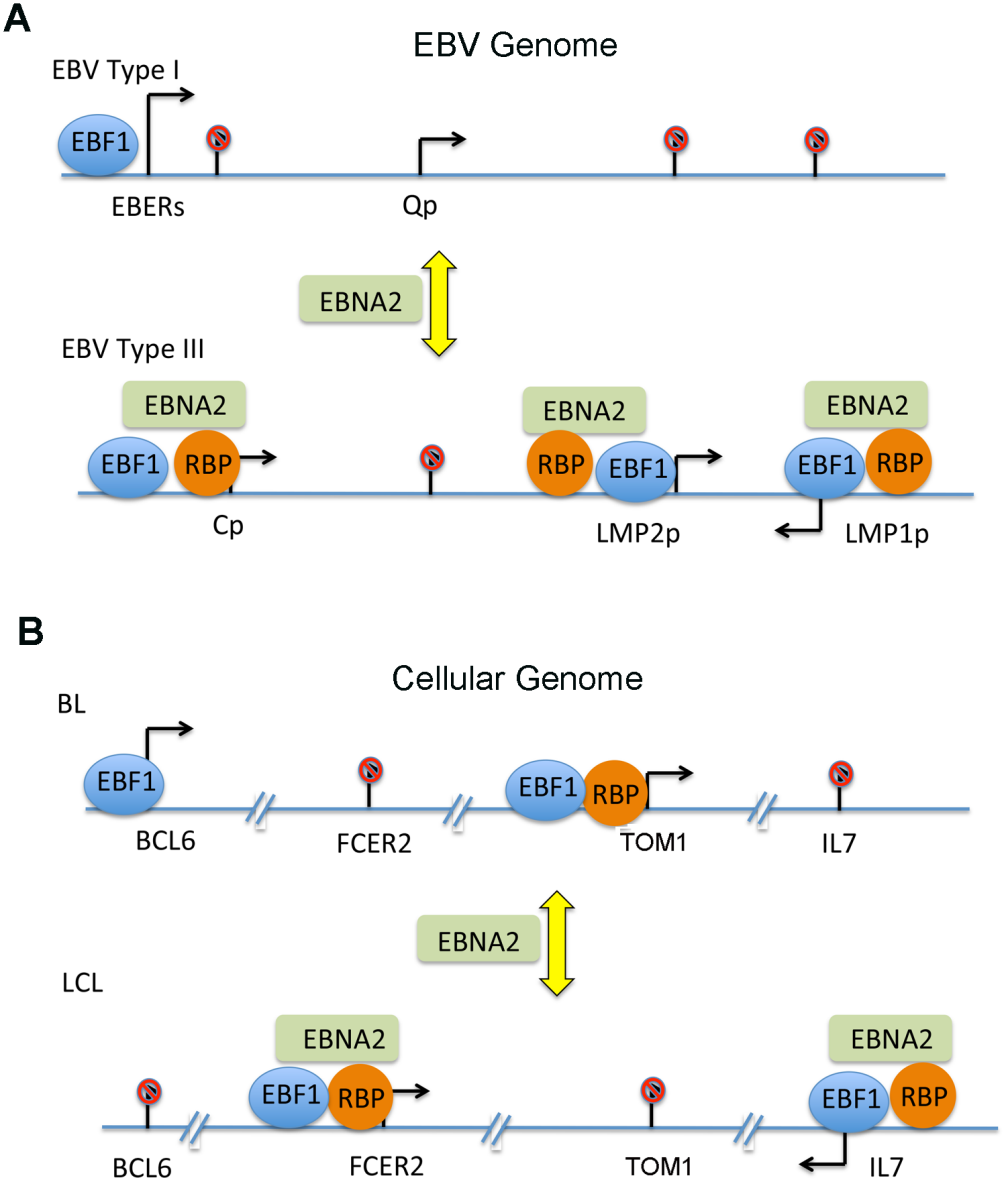
Model of EBNA2-induced transcription factor redistribution in chromosome occupancy. EBNA2 is shown to facilitate the formation of new binding sites that are co-occupied by RBP-jκ and EBF1 relative to cells lacking EBNA2.

EBV genomes adopt different gene expression programs that depend on host cell or tumor type, as well as on epigenetic factors [24, 25]. EBV encodes several transcription cofactors, in addition to EBNA2, that remodel viral and host cell gene programs to confer the EBV-induced proliferation and immortalization to resting B-cells. EBNA2 binds to several sequence specific transcription factors, like RBP-jκ and PU.1, and also interacts with epigenetic co-activators, like histone acetyltransferases CBP/p300 and chromatin remodeling factors containing SNF2 [6]. The prevailing model posits that EBNA2 binds to pre-established sequence-specific binding factors bound to their cognate binding sites. EBNA2 is thought to displace co-repressors bound to these sequence specific factors and then form a stable co-activator complex at selected promoters and enhancers to stimulate target gene transcription. While constitutive DNA binding occurs at some chromosomal sites, our findings indicate that EBNA2 can also drive the formation of new chromosome occupancies for factors like RBP-jκ and EBF1. These newly formed sites are commonly co-occupied by RBP-jκ, EBF1 and EBNA2, and correlate with activated chromatin and transcription function (Fig 8).

A recent study concluded that EBNA2 does not fundamentally alter the binding patterns of RBP-jκ or other sequence specific binding factors [18, 26]. These studies compared LCL to resting B-lymphocytes (RBLs) which do not express EBNA2. Upon EBV infection, EBNA2 was found to colocalize only to pre-existing RBP-jκ binding sites, and only increased RBP-jκ binding peak intensity. In this study, EBNA2 was found to function largely through the formation of enhancer-promoter DNA-loop interactions [18]. Our study differs from these previous reports in several important respects. We compare established LCLs to type I BL cells, as well as established LCLs with a conditional EBNA2-ER fusion protein. Our findings indicate that a large percentage of RBP-jκ and EBF1 binding sites are detected only in the presence of EBNA2. It is likely that some of the RBP-jκ and EBF1 sites exist at low levels in the absence of EBNA2 expression in resting B-lymphocytes, and that threshold cutoffs for ChIP may explain some of these differences in interpretation of data. We also found that some non-colocalized EBF1 and RBP-jκ sites are stable in the absence of EBNA2. However, we find numerous EBF1 and RBP-jκ colocalized sites that form selectively and exclusively in the presence of EBNA2. This suggests that the EBNA2 increases the stability and probability of RBP-jκ and EBF1 binding to a specific group of genomic sites and regulatory elements.

Recent genome-wide studies reveal that these EBV-encoded transcriptional modulators have complex and disparate functions on the cellular genome [12, 18–20, 27]. ChIP-Seq analysis of EBNA2 in a type III BL cells (Mutu III) revealed a high-colocalization with the predicted binding sites of RBP-jκ and EBF1 [19]. Our experimental data confirms this computational prediction in type III LCLs. Additionally, EBNA2 has been implicated in both cooperative and competitive interactions with other viral proteins. For example, EBNA3A can compete with EBNA2 at RBP-jκ sites that regulate CXCL10 and CXCL9 [28]. EBNA-LP colocalizes with ~ 29% of EBNA2 sites, many of which were enriched for EBF1 and RBP-jκ [12]. EBNALP is proposed to remove the co-repressor NCoR from RBP-jκ to induce transcription activation at RBP-jκ bound sites. EBNA3A and 3C were shown to colocalize preferentially with BATF and IRF4, and to a lesser extent with RBP-jκ [20, 27]. Additional levels of complexity may also arise from amino acid variations in EBNA2 proteins derived from different EBV strains. Variants of EBNA2 that more efficiently immortalize B-lymphocytes were found to selectively regulate genes with composite motifs for ETS-interferon regulatory factor (IRF) binding motifs [29]. More recently, EBNA3 proteins have been shown to associate with ubiquitin modifying enzymes that could further alter the stability and functionality of interaction partners [30]. These findings highlight the complex and significant role of non-DNA binding co-factors in altering transcription factor interactions, chromatin occupancy, and function.

Context-dependent binding of RBP-jκ in response to factors like EBNA2 and Notch have been predicted based on meta-data analysis of several reports of various EBNA2 and Notch binding patterns [31]. Other studies have found that RBP-jκ binds dynamically in response to Notch signaling [32–34]. In T-lymphoblastic leukemia cells, RBP-jκ binding was enhanced by the conditional expression of Notch 1 [33]. These Notch 1 induced RBP-jκ sites colocalized with histone H3K4me1 and H3K27ac, suggesting these sites function as enhancer regulatory elements. RBP-jκ chromosome occupancy was also enhanced by Notch1 expression in mouse myogenic cells [34]. The modulatory effects of Notch 1 on RBP-jκ binding site selection are similar to what we observe with EBNA2 induced binding of RBP-jκ. EBNA2 does not have identical activity to Notch, and studies suggest that Notch 2 expression can antagonize EBNA2 growth proliferative activity in EBV immortalized B-lymphocytes [35]. Thus, while EBNA2 and Notch share the ability to bind and modulate RBP-jκ, they are likely to have different patterns of chromosome binding sites and gene activation profiles.

EBF1 has been referred to as a ‘pioneer’ factor due to its ability to initiate an early B-cell gene program and activate genes that are otherwise repressed by chromatin [36]. EBF1 binds and regulates both constitutively active and inducible gene targets during stage-specific B-cell differentiation [37]. However, it is also reported that EBF1 requires priming factors to bind to its chromosomal sites, as it is unable to bind B-cell specific sites in non-hematopoietic cells [13, 36]. EBF1 has been shown to interact with several proteins that could facilitate its pioneering activity, including epigenetic modifiers like TET2, which can promote DNA demethylation [38] and BRG1, which can facilitate nucleosome remodeling [39]. Thus, cellular pioneering factors, like EBF1, are likely to require co-factors to facilitate access to epigenetically repressed sites.

Pioneering co-factors, like EBNA2, may function through several mechanisms Recruiting epigenetic modifiers and chromatin remodelers have been demonstrated at several promoter regulatory elements. But perhaps equally significant, are the combinatorial interactions of transcription factors and co-factors that determine the lineage- and cell-specific enhancer priming [40]. EBNA2 may facilitate combinatorial and cooperative interactions to initiate formation of new enhancer and promoter bound transcription factor complexes. EBNA2 may function as a scaffold that stabilizes multiple protein interactions, including cooperative binding between RBP-jκ and EBF1 at some genomic locations. Our DNA affinity binding assays provide partial support for this model (Fig 6). However, we did not observe a stable interaction of EBF1 with either EBNA2 or RBP-jκ in co-IPs, suggesting that the cooperativity occurs at a functional level distinct form physical association. We also observed gene-specific sensitivities to the depletion of either RBP-jκ or EBF1, suggesting that their functions in transcription regulation are non-redundant (Fig 7). Furthermore, we observed some interdependent chromosome occupancy of these factors, which also appeared gene specific (Fig 7). EBNA2 is also known to recruit histone acetyltransferases p300/CBP and chromatin remodeling factors that can further facilitate transcription factor binding in the context of chromatin. Taken together, these findings suggest that factors like EBNA2, RBP-jκ, and EBF1 can functionally interact in a context-dependent manner to stabilize their coordinated chromatin occupancy and transcription enhancement.

An important biological outcome of EBNA2 function is the overall skewing of binding patterns for the overlapping targets of EBF1 and RBP-jκ. In particular, EBNA2 expression correlated with an increase in the total number and peak intensity of RBP-jκ bound sites (Fig 2). This observation from ChIP-Seq analysis is also consistent with the increase steady state protein expression levels for RBP-jκ in EBNA2 positive cells (Fig 1D). These trends may reflect the role of EBNA2 in driving resting B-cells into proliferating and activated B-lymphoblasts. RBP-jκ is thought to have a major role in T-cell development, while having a more nuanced role in B-cell development, like promoting marginal zone B-lymphocyte differentiation [41]. Our findings suggest that EBV type III latency promotes a B-cell marginal zone developmental program, as the number of RBP-jκ binding sites is largely increased.

In conclusion, our findings demonstrate that the EBV-encoded transcriptional co-activator EBNA2 reprograms the chromosome binding patterns of host master regulatory transcription factors RBP-jκ and EBF1. We propose that EBNA2 drives new combinatorial interactions on DNA at promoter and enhancer regulatory elements of genes essential for EBV lymphoblast proliferation and survival.

## Methods

### Cells

The LCL cell line was generated from human B-cells (provided by the Wistar Institute Phlebotomy Core) transformed with Mutu I virus. EBV positive BL cell lines Mutu I, Kem I, and Kem III were obtained from Dr. Jeffrey Sample, Penn State University Hershey Medical School, PA. LCLs, Mutu I, Kem I and Kem III cells, were maintained in RPMI containing 12% FBS and antibiotics (penicillin and streptomycin). EREB 2.5, a lymphoblastoid cell line expressing the estrogen-inducible EBNA2-estrogen receptor (ER) fusion protein [23], was maintained in RPMI containing 12% FBS, antibiotics (penicillin and streptomycin), and 1μM estradiol.

### Chromatin-immunoprecipitation for high throughput sequencing (ChIP-Seq)

ChIP-Seq experiments were performed with 5 x 10^7^ cells per assay with 10 μg of either rabbit anti- EBF1 (EMD Millipore AB10523), RBP-jκ Abcam AB25949), histone H3K4me3 (EMD Millipore 07–473), H3K4me1 (Active Motif 39297), H3K27me3 (Active Motif 39155) antibodies or control rabbit IgG (Santa Cruz Biotechnology sc-2027). ChIP was performed by as described previously with some modifications [42]. Briefly, crosslinked Mutu I or LCL lysates were sonicated to achieve a DNA fragment length of ~100–500 bp, incubated overnight with antibody-coated Dynabeads protein A/G, then washed with ChIP-seq wash buffer (50 mM HEPES, pH 7.5, 500 mM LiCl, 1 mM EDTA, 1% NP-40, 0.7% Na-Deoxycholate, 1x protease inhibitors) for 5 times, then washed once with 50 mM NaCl in TE buffer. Immunoprecipitated DNA was eluted with ChIP-seq elution buffer (50 mM Tris-HCl, pH 8, 10 mM EDTA, 1% SDS), reverse-crosslinked at 65°C, treated with RNase A (0.2 mg/ml) and proteinase K (0.2 mg/ml), purified with phenol and chloroform, then subjected to qPCR validation. Validated ChIP DNA was isolated by agarose gel purification, ligated to primers, and then subject to Illumina-based sequencing using manufacturers recommendations (Illumina).

### Chromatin immunoprecipitation (ChIP)-qPCR assays

ChIP-qPCR assays were performed as described previously [43]. Quantification of precipitated DNA was determined using real-time PCR and the delta Ct method for relative quantitation (ABI 7900HT Fast Real-Time PCR System). Rabbit IgG, EBF1 and RBP-jκ antibodies used for conventional ChIP assays were same as antibodies used for ChIP-seq. Rabbit CTCF (EMD Millipore 07–729), PU.1 (EMD Millipore 04–1072), PAX5 (Santa Cruz Biotechnology sc-1974x), mouse IgG (Santa Cruz Biotechnology sc-2025), mouse EBNA2 (gift from Paul Farrell, UK) antibodies were also used in ChIP assays. Primers for ChIP assays are listed in Supplement Tables S1 and S2.

### ChIP-reChIP

ChIP-re-ChIP assays were performed as described [44]. Mouse EBNA2 (gift from Paul Farrell, UK), rabbit EBF1 or RBP-jκ antibodies used in ChIP-reChIP were same as antibodies used in conventional ChIP assays.

### RNA extraction and quantitative RT-PCR

RNA was isolated from 2 x 10^6^ cells using RNeasy Kit (Qiagen) and then further treated with DNase I by using DNase treatment and removal kit (Ambion). RT-PCR was performed as previously described [45]. Real-time PCR was performed with SYBR green probe in an ABI Prism 7900 and the delta Ct method for relative quantitation. Primer sequences for RT-PCR are available upon request.

### DNA-affinity purification

DNA-affinity purification was performed as previously described [46]. Soluble nuclear extract fractions were obtained from EREB cells cultured with or without 1μM estradiol for 24 hours. The ~400 bp sequence from the center of EBF1/RPB-jκ peaks at IL7, HES1, or LMP1 loci were cloned into pCR-blunt II-TOPO vector (Invitrogene). The ~500 bp sequence from EBV Qp region (EBV 49712–50250) was cloned into pBSKII. The ~ 400 bp sequence from the center of EBF1/RBPjκ peaks at TOM1 was cloned into pUCIDT vector (IDT). DNA fragments were PCR amplified from their respective plasmid templates using a biotinylated forward primer. The biotinylated PCR fragments were coupled to M-280 streptavidin Dynabeads. The coupled beads were washed and incubated with soluble nuclear extract for 1 hour. The bound proteins and beads were washed three times with D100 buffer (20 mM HEPES, pH 7.9, 20% glycerol, 0.2 mM EDTA, 0.05% NP-40, 100 mM KCl, 1 mM phenylmethylsulfonyl fluoride, 1 mM DTT, and 1x protease inhibitors). The bound proteins were eluted from the beads using 2 x Laemmli buffer and heated at 95°C for 10 minutes, loaded on an 8–16% SDS-PAGE, then analyzed by Western blotting.

### Antibodies used in western blotting

Rabbit polyclonal anti-EBF1 (EMD Millipore AB10523), RBP- jκ (Abcam AB25949), CTCF (EMD Millipore 07–729), PARP-1 (Alexis 210-302-R100), GAPDH (Cell signaling 2118); Goat polyclonal anti-EBF1 (R&D System AF5165); Sheep polyclonal anti-EBNA3C (Exalpha F125P); Rat monoclonal anti-EBNA2 (EMD Millipore MABE8); Mouse monoclonal anti-LMP1 (DAKO M0897); Actin-Peroxidase antibody (Sigma A3854).

### Lentiviral transduction

pLKO.1 vector-based shRNA construct for EBF1 (TRCN0000013831) or RBPjκ (TRCN0000016203) was obtained from Open Biosystems. shControl were generated in pLKO.1 vector with target sequence 5′-TTATCGCGCATATCACGCG-3′. Lentiviruses were produced by the use of envelope and packaging vectors pMD2.G and pSPAX2 as described previously [47]. Mutu I or LCL cells were infected with lentiviruses carrying pLKO.1-puro vectors by spin-infection at 450 g for 90 minutes at room temperature. The cell pellets were resuspended and incubated in fresh RPMI medium, then treated with 2.5 μg/ml puromycin at 48 hr after the infection. The RPMI medium with 2.5 μg/ml puromycin was replaced every 2 to 3 days. The cells were collected after 7 days of puromycin selection, then subject to Western blotting, RT-PCR, ChIP and cell viability assays.

### Cell viability assay

48 hours after lentivirus infection, 96-well assay plate was set up with 10^4^ cells in 100 μL complete RPMI medium with 2.5 μg/ml puromycin in each well. The transduced cells were cultured for 5 days then 10 μl of 0.5 mM resazurin (Sigma) solution were added to each well. The plate was incubated in cell culture incubator for 3–4 hours then read for fluorescence at 560/590 nm. The relative cell viability of shEBF1 transduced cells was presented as a percentage relative to shCtrl.

### Genomic data processing

All ChIP-seq tags for EBNA2, EBF1, RBP-jκ, histone H3K4me3, H3K4me1, H3K27me3, BATF, and JunD were aligned to the human genome hg19 using Bowtie [48] with options ‘-v 2 -m 1 –best–strata’ and all of the redundant tags were removed before downstream analysis. All ChIP-seq data to the human genome were normalized to 10 reads per million mapped reads (RPM). From ENCODE [49], we used ChIP-seq data for BATF (GSM803538) and JunD (GSM754331) in GM12878. For comparison we used ChIP-seq data for EBNA2 in LCL (GSE29498) and Mutu III (GSE47629, sample ID GSM1153765). We also used EBNA3C (GSE52632), and EBNA-LP (GSE49338) in LCL and EBNA3C in Mutu III (GSE47629, sample ID GSM1153766). Peak calling was performed using the findPeaks command in Homer [40]. After initial calling, all of the peaks were resized to 200 bp; the 1 reads per million (RPM) cutoff was applied for EBNA2, EBF1, RBP-jκ to select strong peaks. Specific peaks were defined as having at least a four-fold difference in enrichment within a 200 bp region between the two cell populations. The remaining peaks were defined as common peaks. To compare the ChIP occupancy at cell-type specific peaks, paired t-test was applied. De novo motif finding on the EBNA2 peaks was performed using the findMotifsGenome command in Homer [40]. In the visualization of heatmaps, normalized reads were obtained around the peaks (+/- 4kbp) with a 20bp resolution.

### Gene expression microarrays

RNA samples extracted from three independently cultured Mutu I or LCL cells then further treated with DNase I by using DNase treatment and removal kit (Ambion). RNA quality was determined using the Bioanalyzer (Agilent). Only samples with RIN numbers >7.5 were used for further studies. Equal amounts (400ng) of total RNA was amplified as recommended by Illumina and hybridized to the HumanHT-12 v4 human whole genome bead arrays. Illumina BeadStudio v.3.0 software was used to export expression levels and detection p-values for each probe of each sample. Arrays were quantile-normalized and filtered to remove probes with a detection p-value>0.05 in all samples. Expression level comparisons between the two cell lines were done using two sample t-test and correction for multiple testing to estimate False Discovery Rate (FDR) was performed [50]. FDR<5% genes were considered significant unless stated otherwise. Gene set enrichment analyses were performed using QIAGEN’s Ingenuity Pathway Analysis software (IPA, QIAGEN Redwood City,www.qiagen.com/ingenuity) and only enrichments from “Diseases & Functions” results that passed p<0.001 and Z-score>2 for predicted function activation were reported. Additionally, genes uniquely occupied by EBF1/RBP-jκ/EBNA2 in LCL within 10kb from TSS and significantly over-expressed vs Mutu I at least 1.2 fold were analyzed using IPA’s “Regulator Effect” analysis option to generate a network of predicted affected cellular functions. Differences in proportions of up vs down-regulated or up-regulated vs unchanged genes between classes of occupied genes were tested using Fisher Exact Test. Enrichment values for heatmap with different fold change/distance to TSS windows were calculated using genes significantly different between LCL and Mutu I at nominal p<0.05. Enrichment p-values were estimated by Fisher Exact Test and adjusted for multiple testing with Bonferroni correction. Results with adjusted p<0.05 were considered significant. Windows for any TSS distance X were log_10_-scaled by using formula [10^(log_10_X-0.5) to 10^(log_10_X+0.5)], e.g for 10kb from TSS, a window of [3.16kb to 31.6kb] was generated. TSS distance X for the plot varied from log_10_X = 2 to log_10_X = 5.2 using step of 0.1.

## Supporting Information

S1 Fig Organization of EBF1 and RBP-jκ binding sites at EBV genome sites in LCL and Mutu I cells. **(A)** Magnified view of ChIP-Seq peaks mapped to EBV genome for EBF1, RBP-jκ, and EBNA2 at OriP and Cp regions (left) or LMP2A/ LMP1 promoter regulatory regions (right). **(B)** Sequence analysis of EBF1 and RBP-jκ peak centers and consensus binding sites at Cp, LMP1, and LMP2A.
(TIF)

S2 Fig Motif analysis for top 5 predicted transcription factors binding sites overlapping EBNA2 peaks in LCL cells. (TIF)

S3 Fig **(A)** Cellular ChIP-Seq tracks for genes with LCL-specific co-occupied sites for EBF1, RBP-jκ, and EBNA2. Tracks shown for IL7, HES1, FCER2, and ICA1. **(B)** Cellular ChIP-Seq tracks for genes with Mutu I-specific co-occupied sites for EBF1 and RBP-jκ. Tracks shown for ZNF595, MIR4325, and RNF144B. Primer positions are highlighted in magenta.
(TIF)

S4 Fig Correlation of gene expression with cell specific EBF1 binding peaks. **(A)** Number of genes with RNA-expression differences between LCL (L) and Mutu I (M) were calculated for genes with cell-type specific EBF1 peaks. Genes with EBF1-specific binding sites in LCL were 94.5% likely to have greater transcription levels in LCL. **(B)** Heat map of the top 20 genes with LCL (red) or Mutu I (blue)-specific gene expression and cell-type specific EBF1 binding sites near the TSS. **(C)** RT-qPCR analysis for genes with cell-type specific binding for EBF1 in LCL (red), or for Mutu I (blue). **(D)** Functions enriched and predicted to be activated by genes with cell-specific EBF1 binding and transcription in LCL.
(TIF)

S5 Fig Effect of EBNA2 depletion on EBF1 and RBP-jκ at sites lacking EBNA2. Cells were treated as in main text Fig 5. ChIP assays for EBF1 (**A**), RBP-jκ (**B**), or EBNA2 (**C**) are shown for EREB2.5 cells treated with estradiol (blue), or 24 (pink), or 48 hrs (red) after withdrawal of estradiol. ChIP was assayed for sites associated with genes indicated below each bar graph. Asterisk indicates p < 0.05.
(TIF)

S6 Fig Cell cycle profile for EREB cells before and after estradiol treatment to inhibit EBNA2. EREB2.5 cells treated continuously with estradiol (E2) or at 24 (middle) or 48 hrs (lower panel) after estradiol removal. FACS intensity after propidium iodide incorporation shown in the X-axis and cell number is shown in the Y-axis.
(TIF)

S7 Fig EBNA2 inducible binding of EBF1 and RBP-jκ. EREB2.5 cells were depleted of EBNA2 by withdrawal of estradiol for 72 hrs (grey), followed by re-addition of estradiol for 72 hrs (black). Cells were then subject to ChIP assays for EBF1 (top panels) or RBP-jκ (lower panels). Cellular binding sites for EBNA2 co-occupied sites is shown in left panel, non-EBNA2 co-occupied sites (middle panel), and viral genome sites (right panel). Asterisk indicates p < 0.05.
(TIF)

S8 Fig Co-immunoprecipitation assays. MutuI (M) or LCL (L) cell extracts were subject to IP with control IgG, anti-EBF1, or anti-RBP-jκ, and then assayed by Western blot for EBNA2, EBF1, or RBP-jκ as indicated. Input represents 2% of the total starting lysate for IP. Arrow indicates the RBP-jκ band above the background cross-reacting IgG heavy chain band.
(TIF)

S9 Fig Sequence of EBNA2, RBP-jκ, EBF1 co-occupied sites. Sequence organization of the consensus RBP-jκ and EBF1binding sites in LMP1, IL7, and HES1 promoters used for DNA-affinity assays shown in Fig 6.
(TIF)

S1 Table List of primers used for ChIP-qPCR for EBV genome. (DOCX)

S2 Table List of primers used for ChIP-qPCR for human genome. (DOCX)

S3 Table Entrez ID for genes mentioned in text. (DOCX)
