# Supplementary figures and images for: EBNA2 Drives Formation of New Chromosome Binding Sites and Target Genes for B-Cell Master Regulatory Transcription Factors RBP-jκ and EBF1
Source: PLoS Pathog. 2016 Jan 11;12(1):e1005339. doi: 10.1371/journal.ppat.1005339 (PMC4709166; doi:10.1371/journal.ppat.1005339)

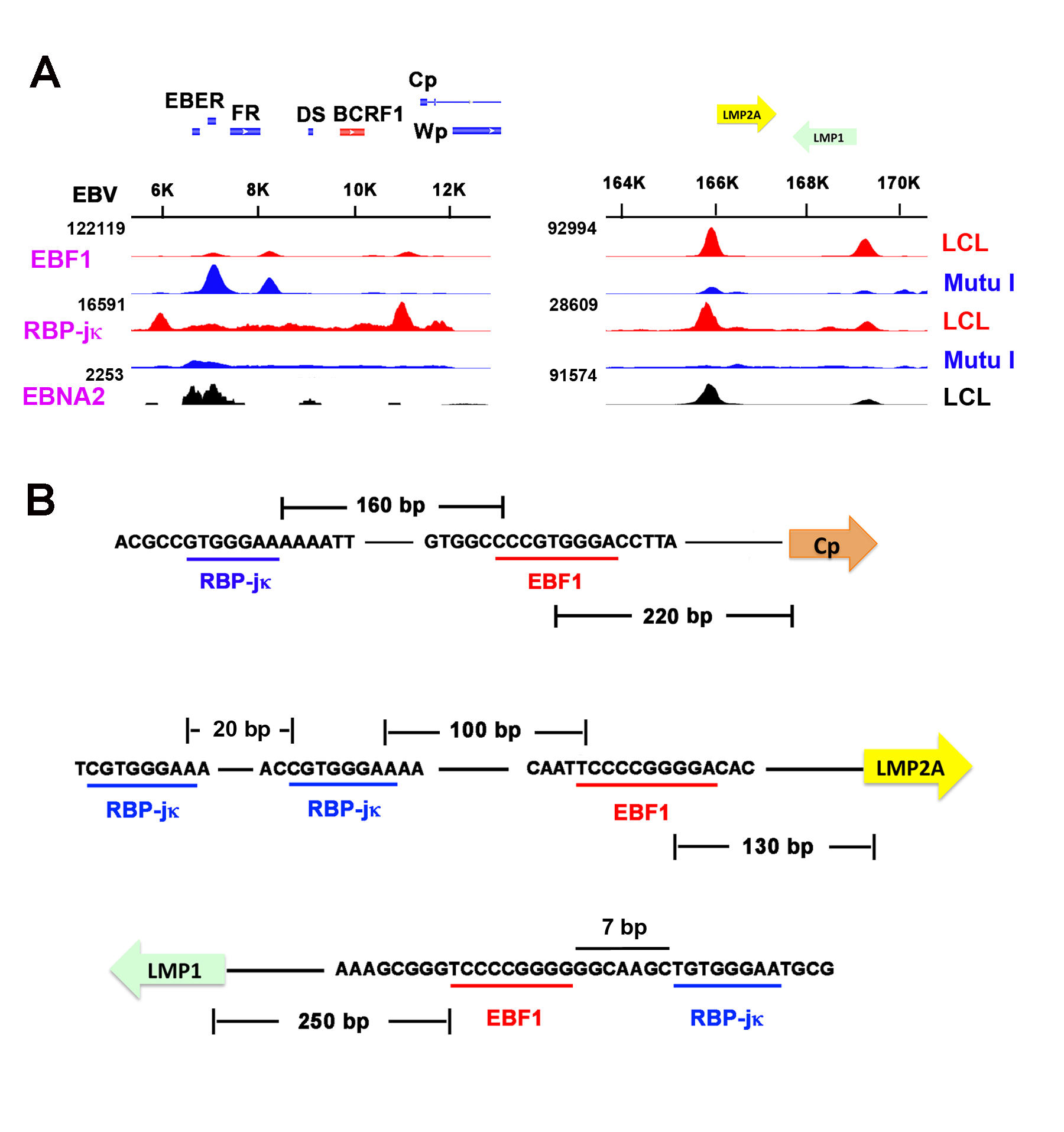

Supplement: S1 Fig — (A) Magnified view of ChIP-Seq peaks mapped to EBV genome for EBF1, RBP-jκ, and EBNA2 at OriP and Cp regions (left) or LMP2A/ LMP1 promoter regulatory regions (right). (B) Sequence analysis of EBF1 and RBP-jκ peak centers and consensus binding sites at Cp, LMP1, and LMP2A. (TIF) [file ppat.1005339.s001.tif]

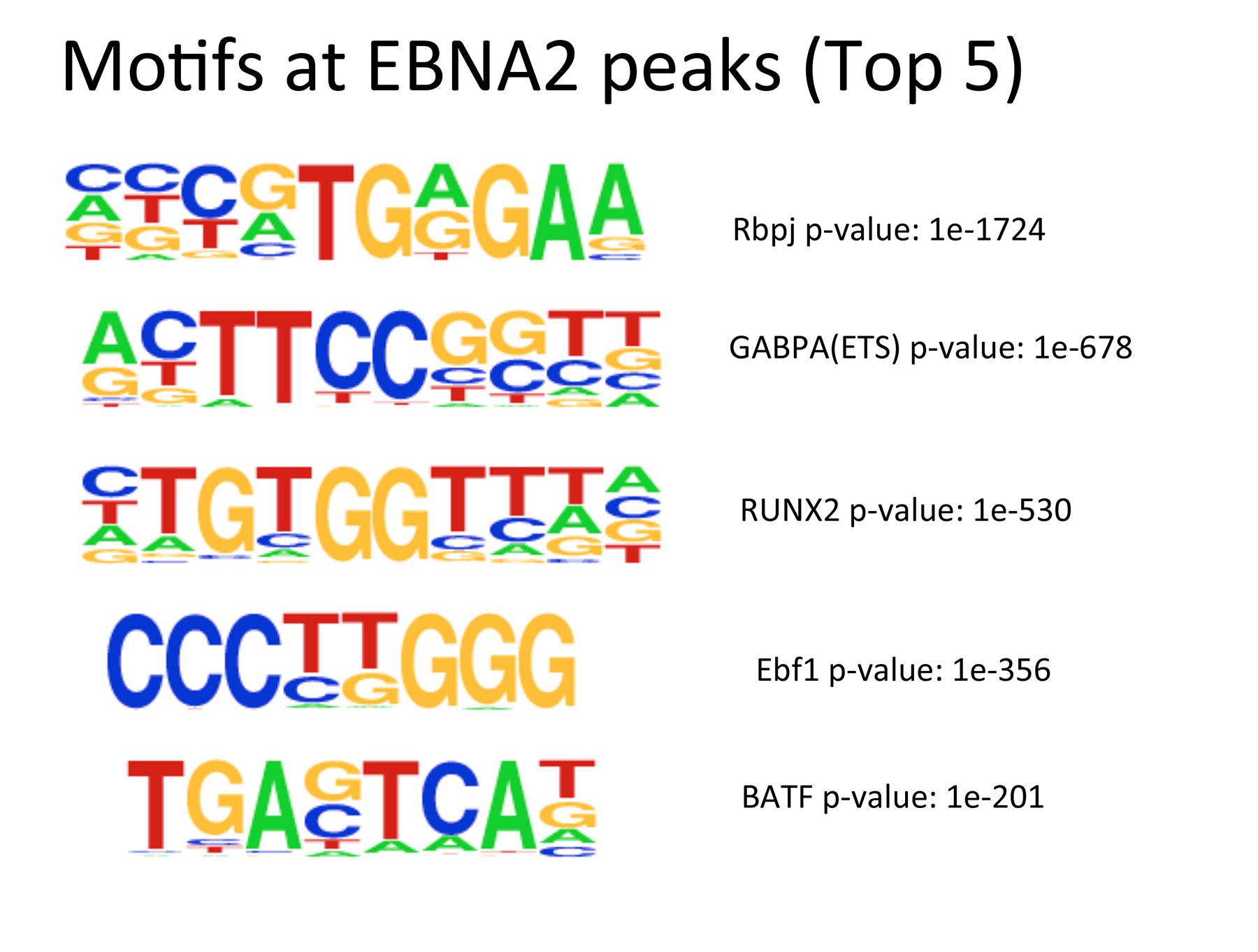

Supplement: S2 Fig — (TIF) [file ppat.1005339.s002.tif]

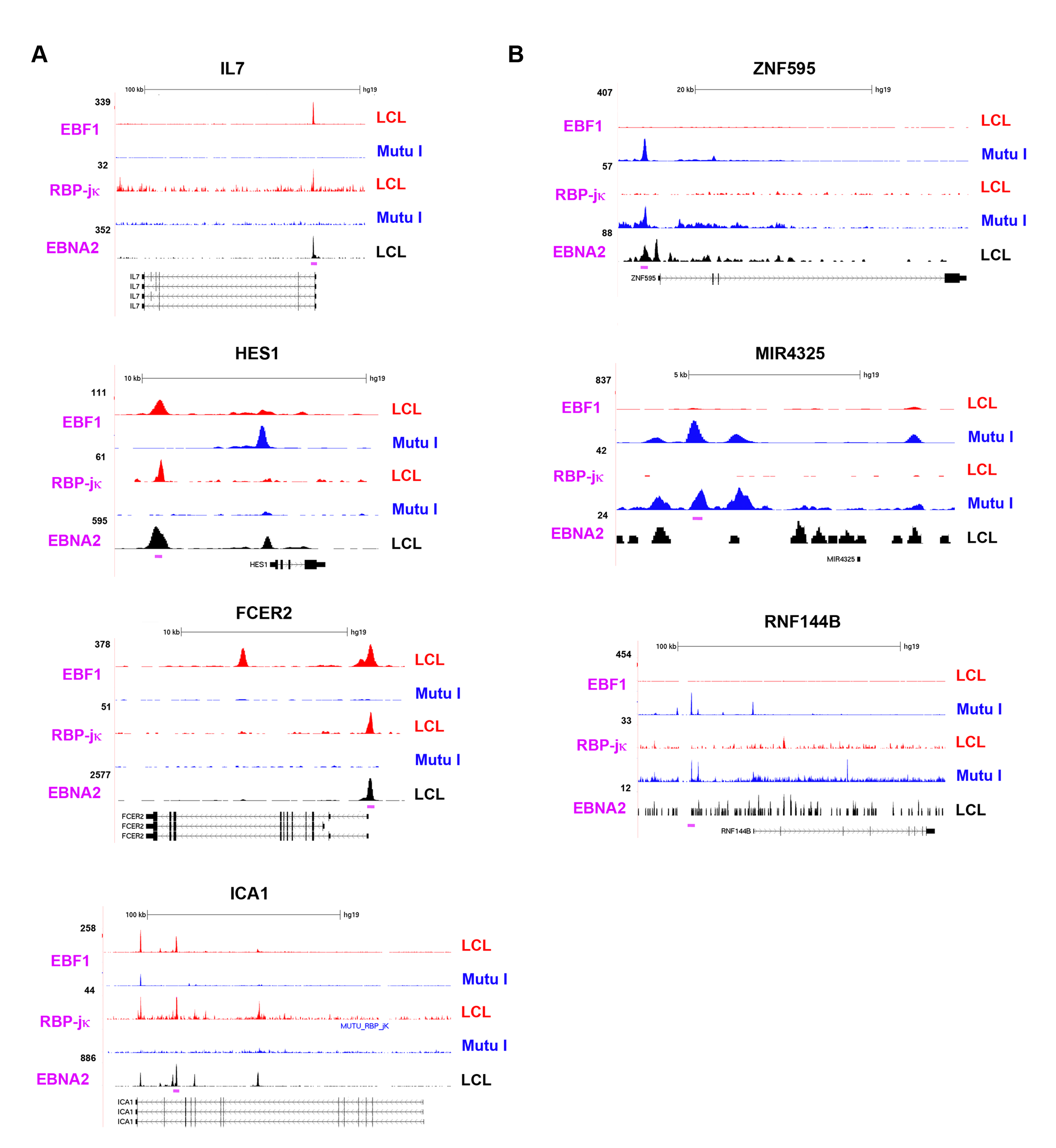

Supplement: S3 Fig — (A) Cellular ChIP-Seq tracks for genes with LCL-specific co-occupied sites for EBF1, RBP-jκ, and EBNA2. Tracks shown for IL7, HES1, FCER2, and ICA1. (B) Cellular ChIP-Seq tracks for genes with Mutu I-specific co-occupied sites for EBF1 and RBP-jκ. Tracks shown for ZNF595, MIR4325, and RNF144B. Primer positions are highlighted in magenta. (TIF) [file ppat.1005339.s003.tif]

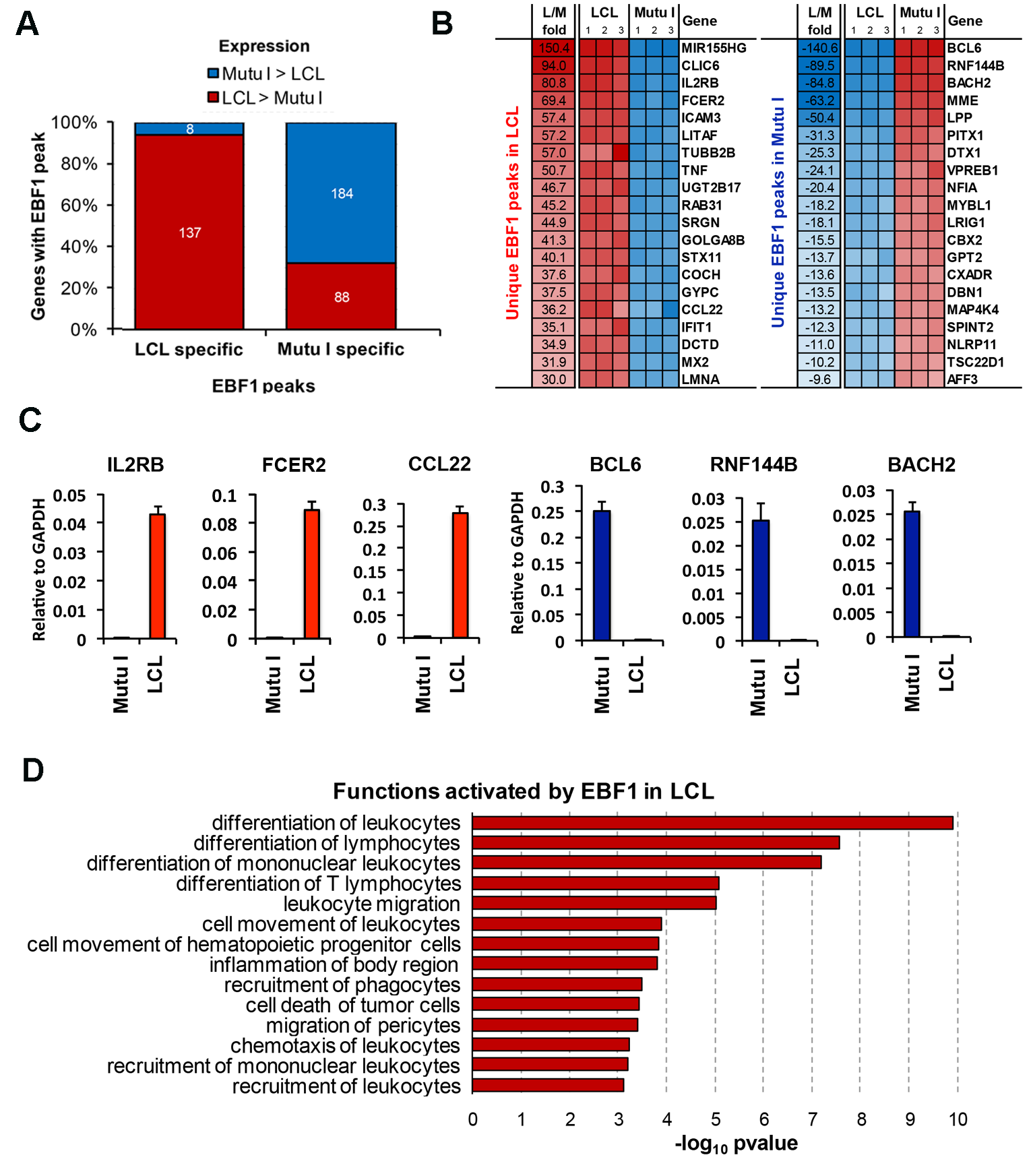

Supplement: S4 Fig — (A) Number of genes with RNA-expression differences between LCL (L) and Mutu I (M) were calculated for genes with cell-type specific EBF1 peaks. Genes with EBF1-specific binding sites in LCL were 94.5% likely to have greater transcription levels in LCL. (B) Heat map of the top 20 genes with LCL (red) or Mutu I (blue)-specific gene expression and cell-type specific EBF1 binding sites near the TSS. (C) RT-qPCR analysis for genes with cell-type specific binding for EBF1 in LCL (red), or for Mutu I (blue). (D) Functions enriched and predicted to be activated by genes with cell-specific EBF1 binding and transcription in LCL. (TIF) [file ppat.1005339.s004.tif]

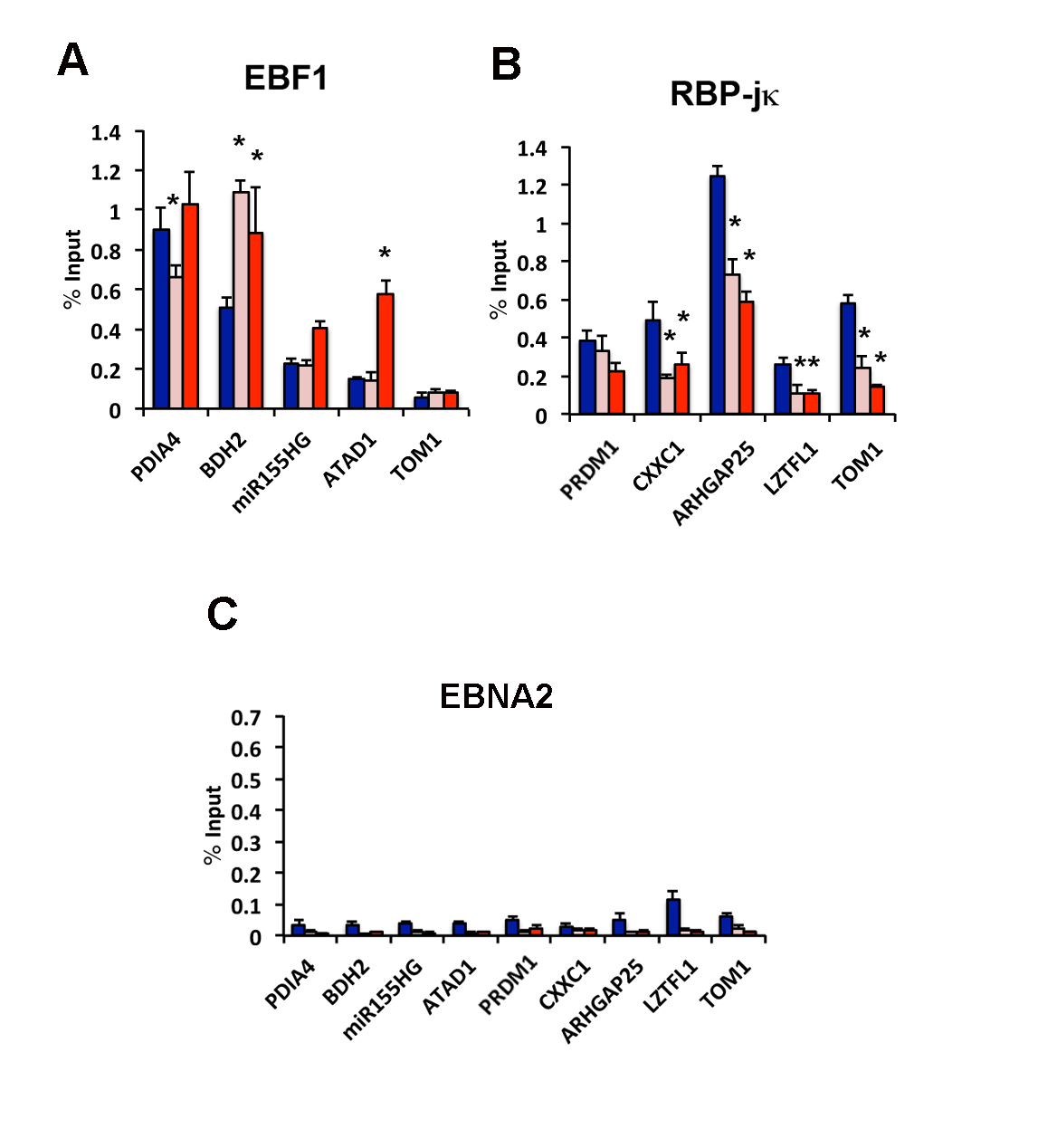

Supplement: S5 Fig — Cells were treated as in main text Fig 5. ChIP assays for EBF1 (A), RBP-jκ (B), or EBNA2 (C) are shown for EREB2.5 cells treated with estradiol (blue), or 24 (pink), or 48 hrs (red) after withdrawal of estradiol. ChIP was assayed for sites associated with genes indicated below each bar graph. Asterisk indicates p < 0.05. (TIF) [file ppat.1005339.s005.tif]

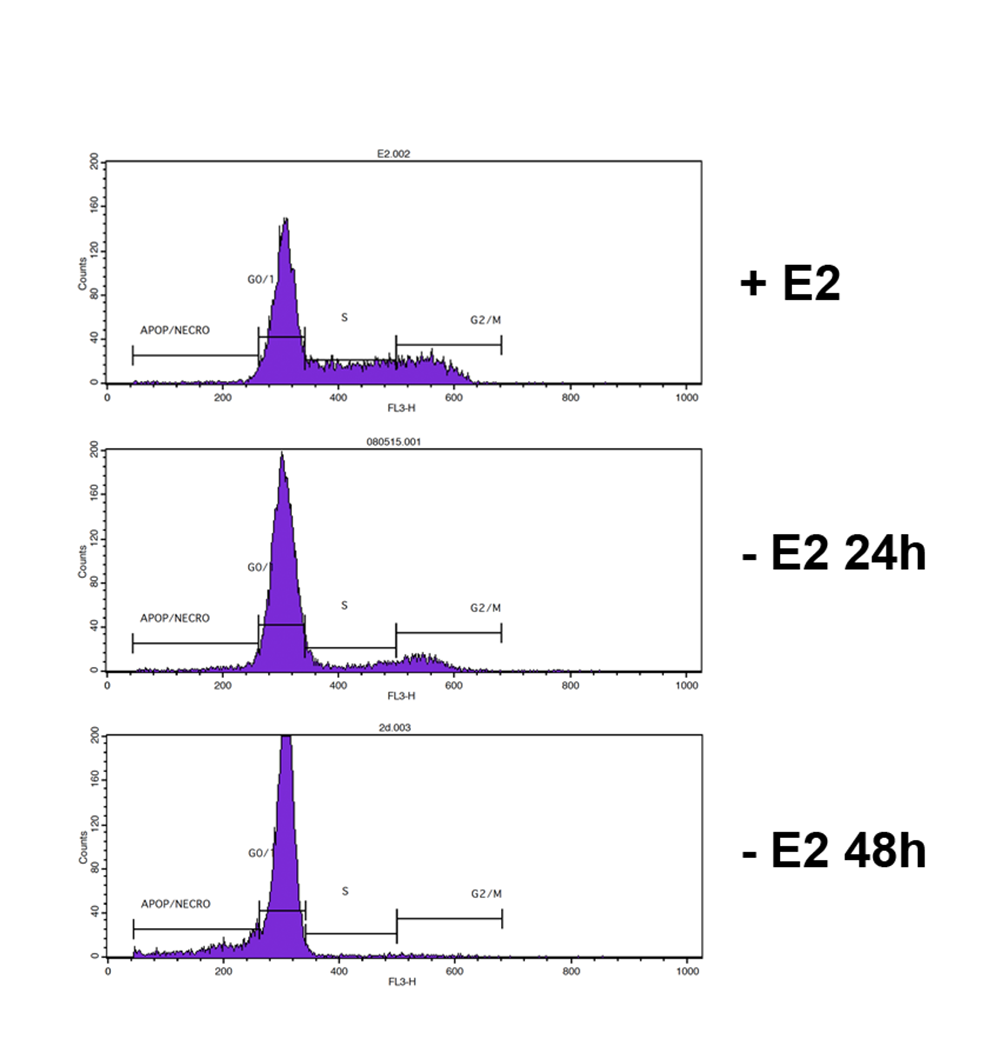

Supplement: S6 Fig — EREB2.5 cells treated continuously with estradiol (E2) or at 24 (middle) or 48 hrs (lower panel) after estradiol removal. FACS intensity after propidium iodide incorporation shown in the X-axis and cell number is shown in the Y-axis. (TIF) [file ppat.1005339.s006.tif]

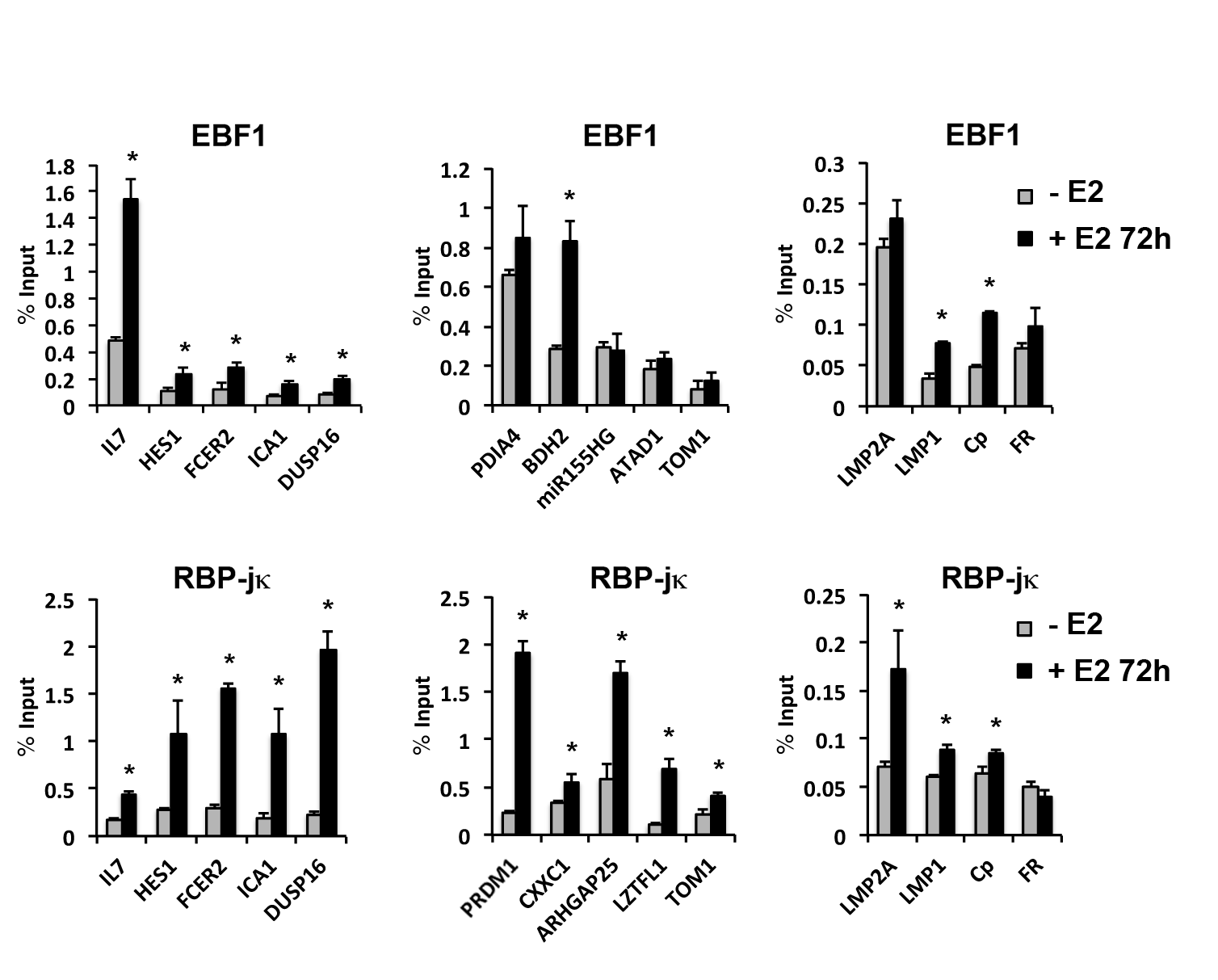

Supplement: S7 Fig — EREB2.5 cells were depleted of EBNA2 by withdrawal of estradiol for 72 hrs (grey), followed by re-addition of estradiol for 72 hrs (black). Cells were then subject to ChIP assays for EBF1 (top panels) or RBP-jκ (lower panels). Cellular binding sites for EBNA2 co-occupied sites is shown in left panel, non-EBNA2 co-occupied sites (middle panel), and viral genome sites (right panel). Asterisk indicates p < 0.05. (TIF) [file ppat.1005339.s007.tif]

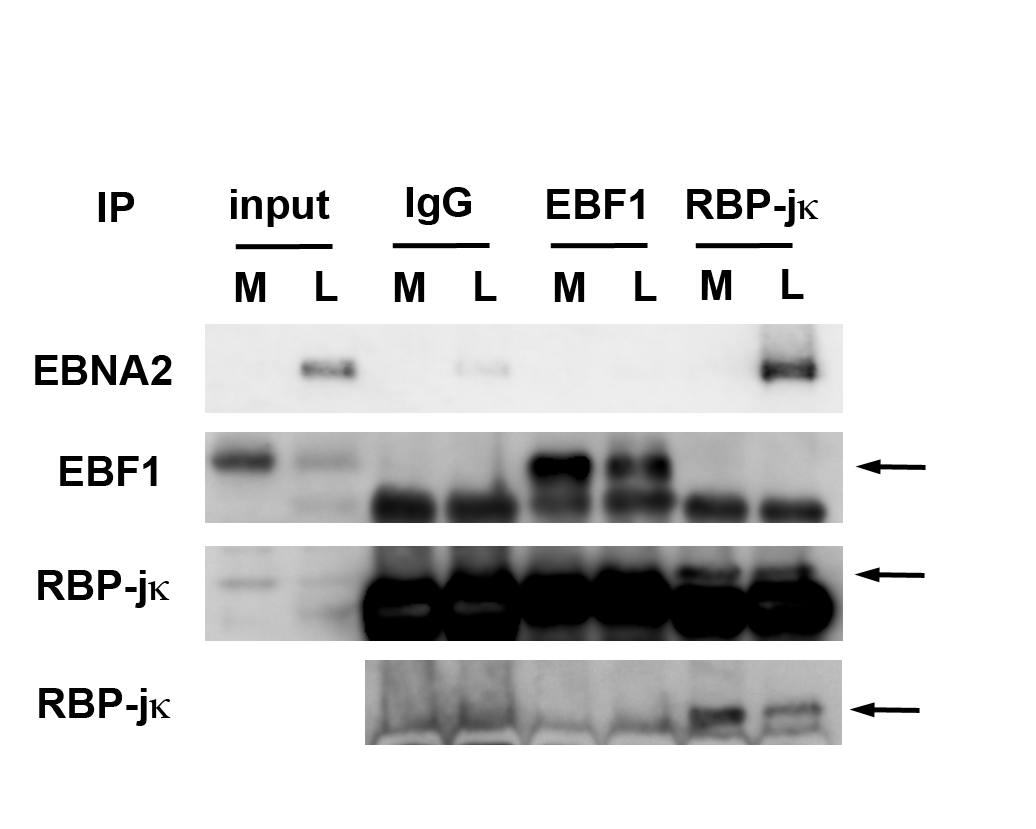

Supplement: S8 Fig — MutuI (M) or LCL (L) cell extracts were subject to IP with control IgG, anti-EBF1, or anti-RBP-jκ, and then assayed by Western blot for EBNA2, EBF1, or RBP-jκ as indicated. Input represents 2% of the total starting lysate for IP. Arrow indicates the RBP-jκ band above the background cross-reacting IgG heavy chain band. (TIF) [file ppat.1005339.s008.tif]

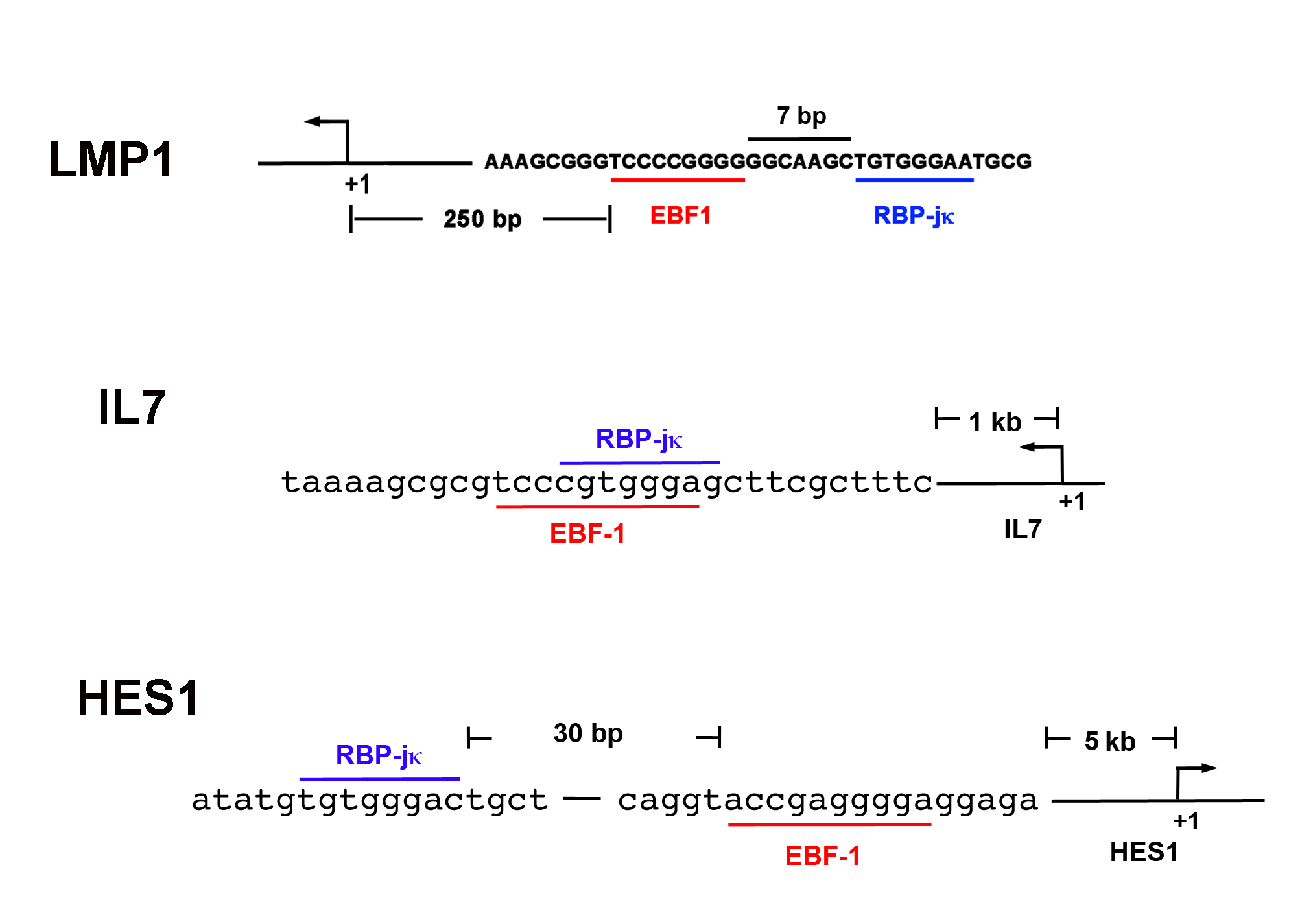

Supplement: S9 Fig — Sequence organization of the consensus RBP-jκ and EBF1binding sites in LMP1, IL7, and HES1 promoters used for DNA-affinity assays shown in Fig 6. (TIF) [file ppat.1005339.s009.tif]
