# Supplementary material for: EBNA2 Drives Formation of New Chromosome Binding Sites and Target Genes for B-Cell Master Regulatory Transcription Factors RBP-jκ and EBF1
Source: PLoS Pathog. 2016 Jan 11;12(1):e1005339. doi: 10.1371/journal.ppat.1005339 (PMC4709166; doi:10.1371/journal.ppat.1005339)
Supplement: S1 Table — (DOCX) [file ppat.1005339.s010.docx]

**Table S1. ChIP primers for EBV genome**

| AAATTGGGTGACCACTGAGGGAGT | Qp_5' |
| --- | --- |
| ATAGCATGTATTACCCGCCATCCG | Qp_3' |
| TATACGAAGAAGCGGGCAGAGGAA | LMP2A_CTCF_BS_5' |
| TGACCTGTTGTCCCTGAGATGTGA | LMP2A_CTCF_BS_3' |
| GCGCCGCGGTTTCAG | LMP2A_5' |
| TTACGCCCCAGCAAGCTT | LMP2A_3' |
| ACGTCAGAGTAACGCGTGTTTC | LMP1_5' |
| GCAGACCCCGCAAATCC | LMP1_3' |
| CAGCGACCTCGTGAATATGA | FR_5' |
| AAACCACTTGCCCACAAAAC | FR_3' |
| ACGGTTCGCTACATCAAACA | EBER_5' |
| GGGAAGCCTCTCTTCTCCTC | EBER_3' |
| GGCGGGAGAAGGAATAACG | Cp_5' |
| CTTGAGCTCTCTTATTGGCTATAATCC | Cp_3' |
