# Supplementary material for: EBNA2 Drives Formation of New Chromosome Binding Sites and Target Genes for B-Cell Master Regulatory Transcription Factors RBP-jκ and EBF1
Source: PLoS Pathog. 2016 Jan 11;12(1):e1005339. doi: 10.1371/journal.ppat.1005339 (PMC4709166; doi:10.1371/journal.ppat.1005339)
Supplement: S2 Table — (DOCX) [file ppat.1005339.s011.docx]

**Table S2. ChIP primers for human genome**

| ACCACCTCTGTAGCTAGCAGGAA | ZNF595_5' |
| --- | --- |
| GGAGCAACATCCTGGCATAAG | ZNF595_3' |
| TCTTTATCTCACTGACATTTCCTTTTG | TOM1_5' |
| ACAAGAGAAACCCTAAAATACCTTACAGA | TOM1_3' |
| GCACCCCCTGCTCTGAATT | RNF144B_5' |
| TGTCAGGACCTGTCTCTTGTTCTC | RNF144B_3' |
| TGCAGCTTGCTCTCTTCTCAAC | PPP1R1B_5' |
| GGCACAAACGAGTACAGCAGTATATG | PPP1R1B_3' |
| CCCCTGTGGGAATTTGCA | PKIG_5' |
| TTGTCTAAATCGAAAGAAACGGTAGA | PKIG_3' |
| TAATTAAGCCATTCGGAGTCTTCA | PRDM1_5' |
| TGTAGGCGGACGGAGTCATT | PRDM1_3' |
| CTATGTTCCAAAAAGCATCTTCCA | PDIA4_5' |
| TCGGGAGGCACAGTGAAAG | PDIA4_3' |
| ACGAAGGTGACTCTTGCATGTG | miR4325_5' |
| AGCTGAACAGATGCTTGGACAA | miR4325_3' |
| GACCAGAGATTGCGCTGGAT | miR155HG_5' |
| TAGGTTTCGAACACCCCTCCTT | miR155HG_3' |
| AAATCGAGAACAGTGATTAAAATGCA | LZTFL1_5' |
| AGCAAACCAAACAGCACTTCTG | LZTFL1_3' |
| CATGGCCTCAGTCCCAAGAG | KCTD17_5' |
| GCCTATGTGTCTGCTACTGCTGAT | KCTD17_3' |
| ACAGCCCTTGCCGGAAA | IL7_5' |
| CTCCGGAAAACCCCATTTTT | IL7_3' |
| TCATGACCTCAGCATAAATTAGAACAC | ICA1_5' |
| GTGCTCTCGTTTTCGGTCTATGT | ICA1_3' |
| TGGCAGGGCAGCTACCA | HES_5' |
| GACTGACTTTCTCACACTCAGATTCC | HES_3' |
| GTCCTTGTCCCCAGCAGAAA | GPR56_5' |
| CCCAGGGTCCAGAGCATCT | GPR56_3' |
| AGAATTGGGTGAGAACTAGAGATGTTAA | FCER2_5' |
| CGCTACCACTCACCTCCTTCA | FCER2_3' |
| GGCTGCAGTGATGTCCTTCA | DUSP16_5' |
| CCCACACATGCCAATAAAGATG | DUSP16_3' |
| CCATGAACTTCCTCATTGTTCCT | CXXC1_5' |
| AGAATCTGAAAATCATCTTGAATGGA | CXXC1_3' |
| TGGAGAATGGGGCCTGAG | CD19_5' |
| AGGTGGCATGGTGGTCAGAC | CD19_3' |
| TAGGATGCAGTGAGCTATGATCATT | BDH2_5' |
| CTGAAACAGGGTTTGTCTTGGTT | BDH2_3' |
| TGCCATAAGTCGTTTACATGTCAGT | ATAD1_5' |
| CTGTAGCATAATGAAAATGAAGAGTAAATG | ATAD1_3' |
| GCTGAGCCTTGCCGTAGTG | ARHGAP25_5' |
| GAGGGTGAAATCCCCATGTG | ARHGAP25_3' |
| GCCATGGTTGTGCCATTACA | Actin_5' |
| GGCCAGGTTCTCTTTTTATTTCTG | Actin_3' |
