# Supplementary material for: EBNA2 Drives Formation of New Chromosome Binding Sites and Target Genes for B-Cell Master Regulatory Transcription Factors RBP-jκ and EBF1
Source: PLoS Pathog. 2016 Jan 11;12(1):e1005339. doi: 10.1371/journal.ppat.1005339 (PMC4709166; doi:10.1371/journal.ppat.1005339)
Supplement: S3 Table — (DOCX) [file ppat.1005339.s012.docx]

**Table S3.**  **Entrez ID for genes mentioned in the text**

| **Entrez ID** | **Gene Name** |
| --- | --- |
| 1879 | EBF1 |
| 3516 | RBPJ (RBP-jκ) |
| 17494192 | EBNA2 |
| 17494242 | EBNA3C |
| 3783750 | LMP1 |
| 17494231 | LMP2A |
| 3574 | IL7 |
| 3280 | HES1 |
| 2208 | FCER2 |
| 3382 | ICA1 |
| 152687 | ZNF595 |
| 100422883 | miR4325 |
| 255488 | RNF144B |
| 80824 | DUSP16 |
| 9601 | PDIA4 |
| 56898 | BDH2 |
| 114614 | miR155HG |
| 84896 | ATAD1 |
| 10043 | TOM1 |
| 639 | PRDM1 |
| 30827 | CXXC1 |
| 9938 | ARHGAP25 |
| 54585 | LZTFL1 |
| 10043 | TOM1 |
| 79734 | KCTD17 |
| 9289 | GPR56 |
| 930 | CD19 |
| 84152 | PPP1R1B |
| 10664 | CTCF |
| 5079 | PAX5 |
| 6688 | SPI1 (PU.1) |
| 2597 | GAPDH |
| 60 | ACTB (Actin) |
| 10538 | BATF |
| 3727 | JunD |
| 142 | PARP1 |
| 1387 | CREBBP (CBP) |
| 2033 | EP300 (p300) |
| 100195999 | CXL10 |
| 4283 | CXCL9 |
| 9611 | NCOR1 |
| 3662 | IRF4 |
| 4851 | NOTCH1 |
| 54790 | TET2 |
| 6597 | SMARCA4 (SNF2, BRG1) |
